# Supplementary material for: Deep learning enables accurate clustering with batch effect removal in single-cell RNA-seq analysis
Source: Nat Commun. 2020 May 11;11:2338. doi: 10.1038/s41467-020-15851-3 (PMC7214470; doi:10.1038/s41467-020-15851-3)
Supplement: Supplementary file 1 — Supplementary Information [file 41467_2020_15851_MOESM1_ESM.pdf]

Supplementary Information

Deep learning enables accurate clustering with batch effect removal in single-cell RNA-seq analysis

Xiangjie Li, Kui Wang, Yafei Lyu, Huize Pan, Jingxiao Zhang, Dwight Stambolian, Katalin Susztak, Muredach P. Reilly, Gang Hu*, and Mingyao Li*

*Correspondence:

Mingyao Li, Ph.D.

mingyao@pennmedicine.upenn.edu

Gang Hu, Ph.D.

huggs@nankai.edu.cn

Supplementary Table 1. Datasets analyzed in this paper 2

Supplementary Table 2. The numbers of hidden layers and nodes in DESC encoder 4

Supplementary Table 3. Default hyperparameters of DESC 4

Supplementary Table 4. Software compared with DESC 5

Supplementary Note 1: Introduction of the methods compared in this paper...................7

Supplementary Note 2: Analysis of the macaque retina 8

Supplementary Note 3: Analysis of the human pancreas data 15

Supplementary Note 4: analysis of the human PBMC data 19

Supplementary Note 5: Analysis of the mouse bone marrow data..................................27

Supplementary Note 6: Analysis of the human monocyte data 28

Supplementary Table 5. P-values for comparing the pseudo-time distributions among the three batches using Kolmogorov-Smirnov test. 31

Supplementary Note 7: Analysis of the mouse brain data with 1.3 million cells.............32

Supplementary References...34

Supplementary Table 1. Datasets analyzed in this paper.

Species	Tissue	Data source	No. of subjects	Cell types (Number of cells)	Sample size	Protocol
Macaque	Retina	Peng et al. (2019)<br>(GSE118480)	2 regions;<br>4 animals;<br>30 samples	BB/GB* (1,815)<br>DB1 (996)<br>DB2 (2,244)<br>DB3a (623)<br>DB3b (2,640)<br>DB4 (2,985)<br>DB5* (3,467)<br>DB6 (658)<br>FMB (4,500)<br>IMB (6,151)<br>OFFx (147)<br>RB (4,076)	30,302 cells	Drop-seq
Human	Pancreas	Grün D et al. (2016) (GSE81076);<br>Muraro et al. (2016) (GSE85241);<br>Lawlor et al. (2017) (GSE86469);<br>Segerstolpe et al. (2015) (E-MTAB-5061)	4 batches	acinar (711)<br>activated_stellate (180)<br>alpha (2,281)<br>beta (1,172)<br>delta (405)<br>ductal (1,065)<br>endothelial (61)<br>epsilon (14)<br>gamma (359)<br>macrophage (24)<br>mast (17)<br>quiescent_stellate (20)<br>schwann (12)	6,321 cells	CelSeq;<br>CelSeq2;<br>Fluidigm C1;<br>SMART-Seq2
Human	PBMC	Kang et al. (2018)<br>(GSE96583)	8 subjects;<br>2 batches	B cells (2,573)<br>CD14+ Monocytes (5,385)	24,679 cells	10X

				CD4 T cells (10,389)<br>CD8 T cells (2,042)<br>Dendritic cells (432)<br>FCGR3A+ Monocytes (1,599)<br>Megakaryocytes (260)<br>NA (6)<br>NK cells (1,993)		
Mouse	Bone marrow	Paul et al. (2016)<br>(GSE72857)	1 batch	Ery (1,095)<br>MEP (167)<br>Mk (68)<br>GMP (216)<br>Baso (369)<br>Mo (559)<br>Neu (186)<br>Eos (9)	2,730 cells	MARS-seq
Human	Monocyte s	Generated by us	1 subject;<br>3 batches	-	10,878 cells	10X
Mouse	Brain	10X website	1 batch	-	1,306,127 cells	10X

Note: All cell types label were given by authors of the original papers. The cell types were identified by complex clustering methods and were verified by known cell type markers.

Supplementary Table 2. The numbers of hidden layers and nodes in DESC encoder.

No. of Cells	No. of hidden layers and 'tol' value	No. of nodes in the 1st hidden layer	No. of nodes in the 2nd hidden layer
>10,000	2 (tol=0.001)	128 (or larger)	32
(5,000,10,000]	2 (tol=0.001)	64	32
(2,000,5,000]	2 (tol=0.005)	64	32
(500,2,000]	1 (tol=0.005)	64	0
<500	1 (tol=0.01)	16	0

Note: The iterative procedure in DESC stops when the proportion of cells that changes cluster assignment between two consecutive iterations is less than tol. Specifically, tol is calculated as $tol = \frac{\#|Y_{curr} \neq Y_{prev}|}{n}$, where Y_{curr} is the cluster id obtained by the maximum cluster assignment probability in the current step, Y_{prev} is the corresponding cluster id in the previous step, n is the total number of cells, and $\#|Y_{curr} \neq Y_{prev}|$is the number of cells in which Y_{curr} does not agree with Y_{prev}.

Supplementary Table 3. Default hyperparameters of DESC.

Parameter	Default value
Activation function	ReLU or Tanh
Kernel initializer	glorot_uniform
Dropout rate	0.2
Optimizer	Stochastic gradient descent
Learning rate	0.01
Batch Size	256
No. of epochs	300

Supplementary Table 4. Software compared with DESC.

Method	Software name	Version	Url	reference
DESC	desc	1.0.0.5	https://github.com/eleozzr/desc	-
Seurat3.0	Seurat	3.0.0	https://github.com/satijalab/seurat	T. Stuart et al., “Comprehensive Integration of Single-Cell Data,” Cell, vol. 177, no. 7, pp. 1888-1902.e21, Jun. 2019
CCA	Seurat	2.3.4	https://github.com/satijalab/seurat	A. Butler, P. Hoffman, P. Smibert, E. Papalexi, and R. Satija, “Integrating single-cell transcriptomic data across different conditions, technologies, and species,” Nature Biotechnology, vol. 36, no. 5, pp. 411–420, May 2018
MNN	Scanpy	1.3.6	https://github.com/theislab/scanpy/	L. Haghverdi, A. T. L. Lun, M. D. Morgan, and J. C. Marioni, “Batch effects in single-cell RNA-sequencing data are corrected by matching mutual nearest neighbors,” Nature Biotechnology, vol. 36, no. 5, pp. 421–427, May 2018
scVI	scvi	0.3.0	https://github.com/YosefLab/scVI	R. Lopez, J. Regier, M. B. Cole, M. I. Jordan, and N. Yosef, “Deep generative modeling for single-cell transcriptomics,” Nature Methods, vol. 15, no. 12, p. 1053, Dec. 2018
BERMUDA	BERMUDA	-	https://github.com/txWang/BERMUDA	T. Wang *et al.*, “BERMUDA: a novel deep transfer learning method for single-cell RNA sequencing batch correction reveals hidden high-resolution cellular subtypes,” *Genome Biology*, vol. 20, no. 1, p. 165, Aug. 2019.
scanorama	scanorama	1.4	https://github.com/brianhie/scanorama	B. Hie, B. Bryson, and B. Berger, “Efficient integration of heterogeneous single-cell transcriptomes using Scanorama,” *Nature*

				Biotechnology, vol. 37, no. 6, p. 685, Jun. 2019.
monocle3	monocle	monocle3 alpha	https://github.com/cole-trapnell-lab/monocle-release/tree/monocle3_alpha	J. Cao et al. “The single-cell transcriptional landscape of mammalian organogenesis”. Nature 566, 496-502, Feb 2019.

Supplementary Note 1: Introduction of the methods compared in this paper.

This paper compared four state-of-the-art methods for scRNA-seq clustering with batch effect removal. Below we describe the parameters used for each method.

Seurat 3.0: This method was developed by Stuart et al. (2019). For each dataset, we used the same number of cells as DESC, and performed analysis following Seurat 3.0's tutorial (https://satijalab.org/seurat/v3.0/immune_alignment.html). Specifically, we selected top 2,000 highly variable genes (nfeatures=2000 in the FindVariableFeatures function) for each batch and other parameters were specified following the tutorial.

CCA: This method was developed by Butler et al. (2018) and implemented in Seurat 2.0. We conducted CCA using the Seurat version 2.3.4 R package. We selected top 2,000 highly variable genes for each batch (the default parameters of CCA in Seurat version 2.3.4), and chose genes with frequency larger than 2. Additionally, the top 20 Canonical Components were selected for alignment.

MNN: This method was developed by Haghverdi et al. (2018), which is a strategy for batch effect correction based on the detection of mutual nearest neighbors (MNNs) in the high-dimensional expression space. We similarly selected top 2,000 highly variable genes for each batch, and then chose genes with frequency larger than 2 in downstream analyses.

scVI: This method was developed by Lopez et al. (2019), which uses stochastic optimization and deep neural networks to aggregate information across similar cells and genes to approximate the distributions that underlie observed expression values, while accounting for batch effect. We used the same highly variable genes as Seurat 3.0, CCA and MNN, and conducted clustering analysis using Louvain's method (Blondel et al. (2008)) with the scVI low-dimensional gene expression representation as the input.

BERMUDA: This method was developed by Wang et al (2019), which firstly applies a graph-based clustering algorithm on each batch individually to detect cell clusters and then, MetaNeighbor, a method based on Spearman correlation, is used to identify similar clusters between batches. An autoencode is subsequently trained to perform batch correction on the code of the autoencoder. The code of the autoencoder is a low-dimensional representation of the original data without batch effects and can be used for further analysis. We used the default parameters unless otherwise stated.

scanorama: This method was developed by Hie et al (2019), which firstly uses singular value decomposition (SVD) for combined datasets to conduct dimension reduction and then identifies shared cell types among all pairs of datasets using a "mutual nearest neighbors" strategy. Then these mutually linked cells form matches that can be leveraged to correct for batch effects and integrate batches together using "panorama" strategy. We used the default parameters unless otherwise stated.

Supplementary Note 2: Analysis of the macaque retina

This dataset was generated by Peng et al. (2019). Molecular Classification and Comparative Taxonomics of Foveal and Peripheral Cells in Primate Retina. Cell 176(5), 1222-1237, https://doi.org/10. 1016/j.cell.2019.01.004.

The original paper has 165,679 cells, including 42,020 retinal ganglion cells (RGCs), 36,268 Non-neuronal cells (NN), 30,302 bipolar cells (BC), 30,236 amacrine cells (AC), 24,707 photoreceptor cells (PR) and 2,146 horizontal cells (HC). But here we only focus our analysis on the 30,302 bipolar cells. The macaque fovea scRNA-seq data matrix can be downloaded from GSE118480 and macaque peripheral single cell RNA-seq data can be downloaded from GSE118852. The 30,302 BC cells include 12 subclusters: IMB, FMB, RB, DB5*, DB4, DB3b, DB2, BB/GB*, DB1, DB6, DB3a, and OFFx. This dataset has three levels of batch indexes, which are macaque_id (the animal ID or subject id), region (fovea or periphery of each macaque), and sample (sample ID, each animal may have multiple replicates).

Cell filtering criteria: 1) we did not filter out any cells because the downloaded data were already prefiltered.

Gene filtering criteria: 1) mitochondrial genes were eliminated; 2) a gene was eliminated if the number of cells expressing this gene is <10.

Data processing: 1) gene expression levels for each cell was normalized using the "*scanpy.api.normalize_per_cell*" function in scanpy with counts_per_cell_after = 10,000; 2) top 1,000 highly variable genes were selected using the "*scanpy.api.pp.filter_genes_dispersion*" function in scanpy; 3) normalized gene expression for the selected top 1,000 highly variable genes was then transformed using log(1+x) transformation with natural logarithm; 4) the expression value was further standardized to a z-score for cells within each batch based on specified batch ID, and the standardized gene expression values were used as input for DESC. After the above filtering and data processing, there were 30,302 cells×1,000 highly variable genes remained in DESC analysis.

DESC analysis: We used two hidden layers for encoder with 128 nodes in the first hidden layer, and 32 nodes in the second hidden layer. Other parameters were set as default values. The final model is 1000-128-32-128-1000.

Remark: This dataset is relatively complex because it contains three different levels of batch: macaque ids, sample ids, and region ids. So for each method, we took macaque id, sample id, region id as the batch, respectively. When batch information is provided, DESC performs expression value standardization within each batch; otherwise, DESC would perform expression value standardization across cells in all batches. Using this dataset, we show the robustness of DESC, that is, it yields accurate clustering result (**Fig. 1e**) and mixes cells from different batches well regardless the definition of batch in the expression standardization step (**Fig. 3a**). In addition, due to memory issue, CCA and Seurat3.0 for this dataset were conducted on CentOS Linux release 7.5.1804 (Core) with Intel(R) Xeon(R) CPU E7-4850 v4 @ 2.10GHz and total 1TB memory.

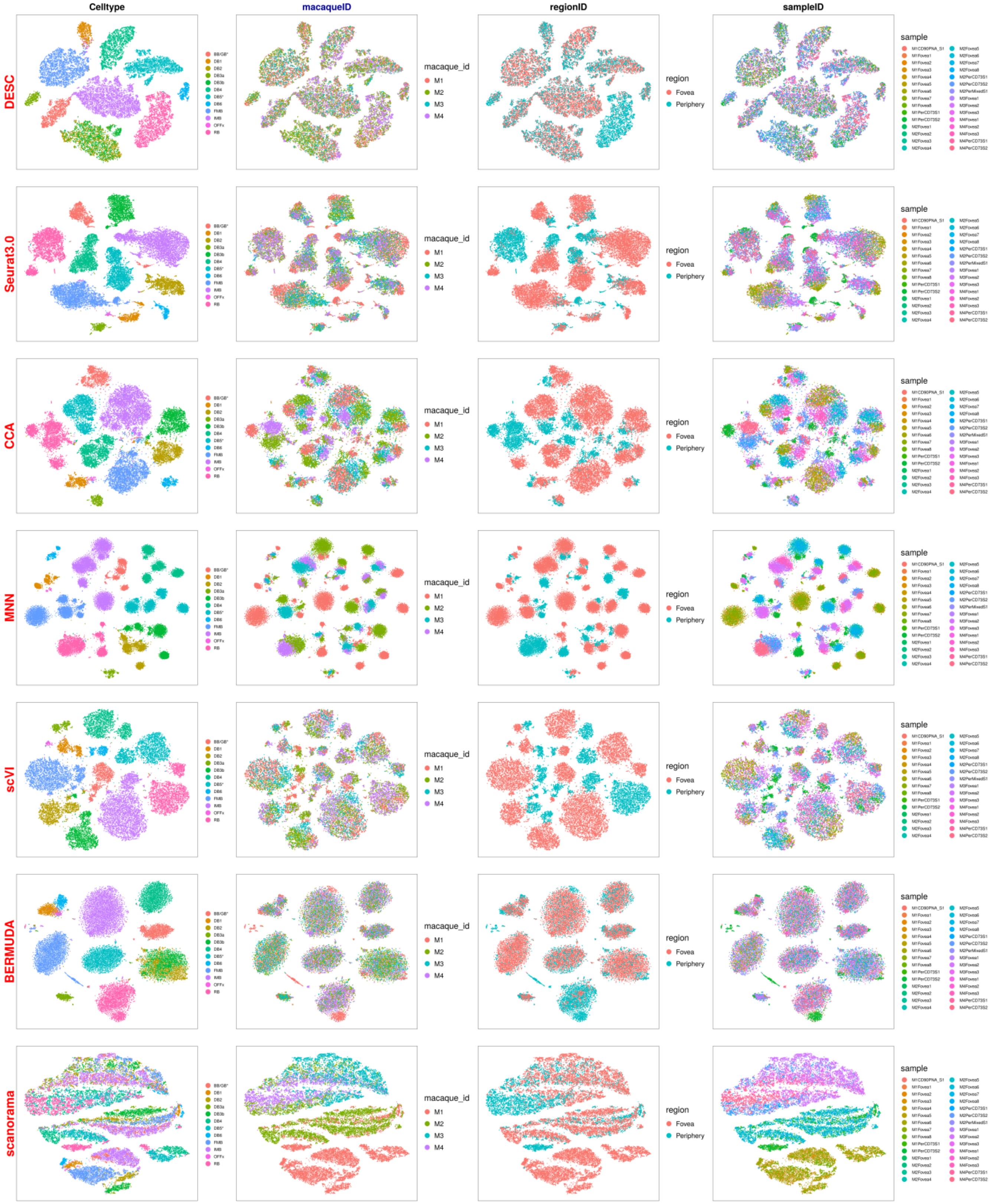

Supplementary Fig 1. t-SNE plots showing batch distribution when taking **macaque id** as the batch definition by different methods. The batch effect removal for each method was done using the provided batch definition, but displayed are the batch distribution for macaque id, region, and sample, respectively. The purpose is to show the impact of batch definition on the mixing of cells at different batch levels. The

first row is the result from **DESC**, which shows that all the batches mix well according to the t-SNE plot. The second row is the result from **Seurat3.0.** The third row is the result from **CCA.** The fourth row is the result from **MNN.** The fifth row is the result from **scVI**. The sixth row is the result from **BERMUDA** with similarity threshold 0.90. The last row is the result from **scanorama.**

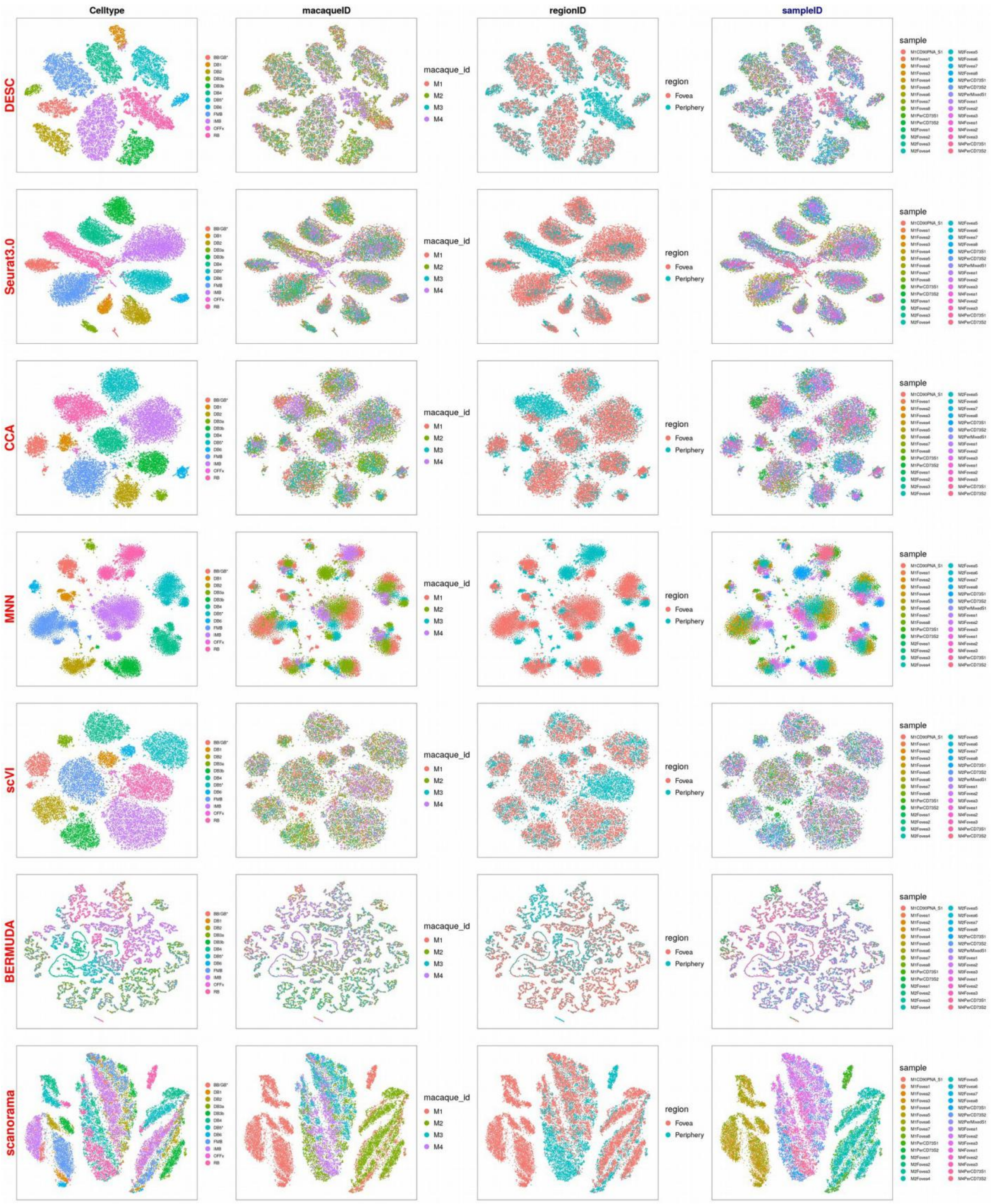

Supplementary Fig 2. t-SNE plots showing batch distribution when taking **region id** as the batch definition by different methods. The batch effect removal for each method was done using the provided batch definition, but displayed are the batch distribution for macaque id, region, and sample, respectively. The purpose is to show the impact of batch definition on the mixing of cells at different batch levels. The

first row is the result from **DESC**, which shows that all the batches mix well according to the t-SNE plot. The second row is the result from **Seurat3.0.** The third row is the result from **CCA.** The fourth row is the result from **MNN.** The fifth row is the result from **scVI**. The sixth row is the result from **BERMUDA** with similarity threshold 0.90. The last row is the result from **scanorama.**

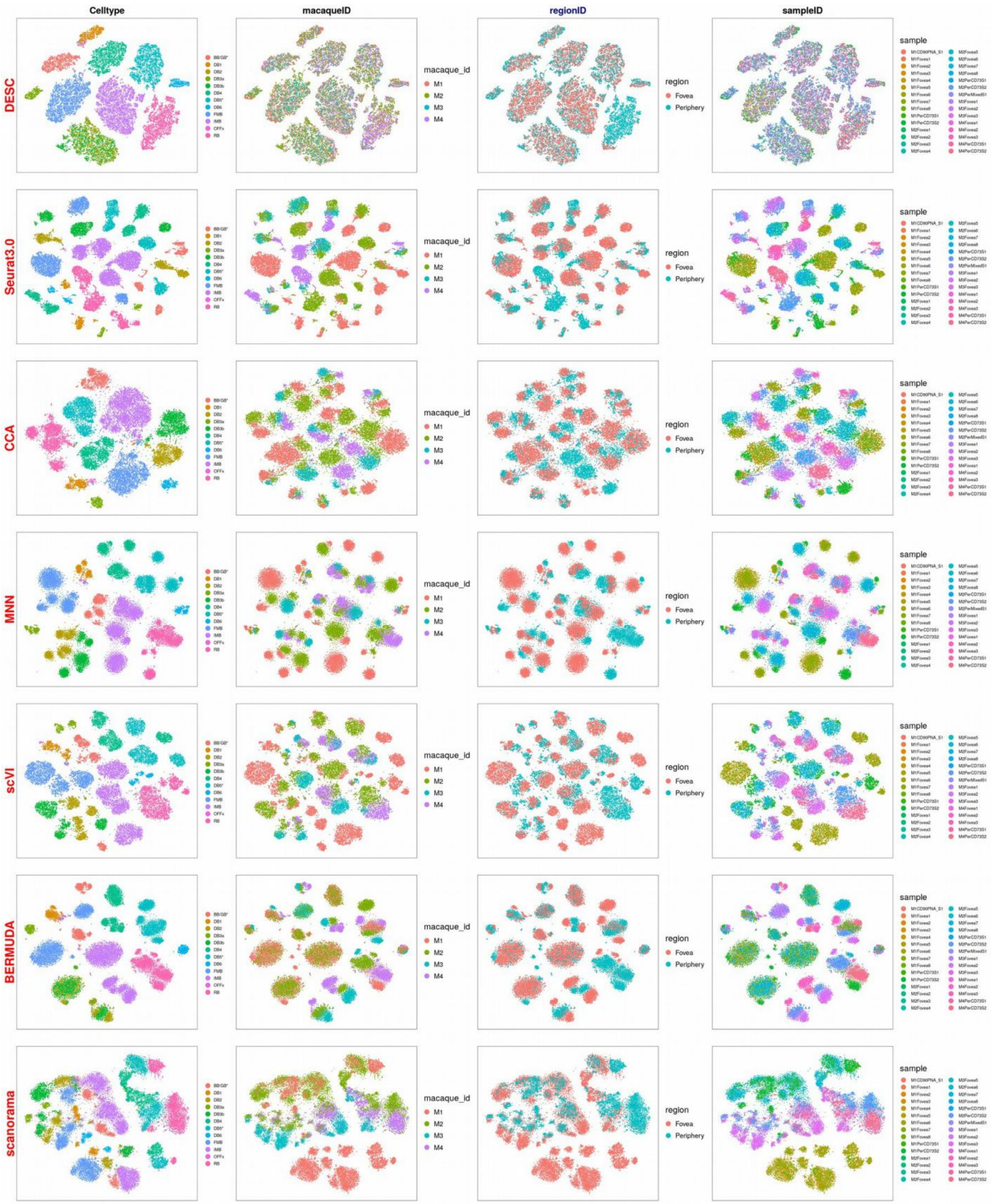

Supplementary Fig 3. t-SNE plots showing batch distribution when taking **sample id** as the batch definition by different methods. The batch effect removal for each method was done using the provided batch definition, but displayed are the batch distribution for macaque id, region, and sample, respectively. The purpose is to show the impact of batch definition on the mixing of cells at different

batch levels. The first row is the result from **DESC**, it can be seen that all the batches mix well according to the t-SNE plot. The second row is the result from **Seurat3.0.** The third row is the result from **CCA.** The fourth row is the result from **MNN.** The fifth row is the result from **scVI**. The sixth row is the result from **BERMUDA** with similarity threshold 0.90. The last row is the result from **scanorama.**

The above three figures (**Supplementary Fig1-Fig3**) indicate that DESC is robust to the definition of batch, even though **sample id** was used as the batch definition when calculating standardized gene expression values of DESC, the cells were still mixed well by macaque id and by region id. However, all other methods are sensitive to the batch definition, and the cells were not mixed well by macaque id and region id.

Supplementary Note 3: Analysis of the human pancreas data

In order to evaluate the performance of DESC for data generated from different scRNA-seq protocols, we analyzed four human pancreatic islet datasets, and compared DESC with six other batch effect removal methods, including Seurat3.0, CCA, MNN, scVI, BERMUDA and scanorama. The four protocols we considered include CelSeq (GSE81076), CelSeq2 (GSE85241), Fluidigm C1 (GSE86469), and SMART-Seq2 (E-MTAB-5061). The combined raw data matrix and associated metadata file can be downloaded from https://www.dropbox.com/s/1zxbn92y5du9pu0/ pancreas_v3_files.tar.gz?dl=1

The combined dataset has 6,321 cells, with 1,004 cells from GSE81076, 2,285 cells from GSE85241, 638 cells from GSE86469, and 2,394 cells from E-MTAB-5061. This combined dataset contains 13 cell types: acinar, activated_stellate, alpha, beta, delta, ductal, endothelia, epsilon, gamma, macrophage, mast, quiescent_stellate and schwann.

Cell filtering criteria: 1) we did not filter out any cells because the downloaded data were already prefiltered.

Gene filtering criteria: 1) mitochondrial genes were eliminated; 2) a gene was eliminated if the number of cells expressing this gene is <10.

Data processing: 1) gene expression levels for each cell was normalized using the "*scanpy.api.normalize_per_cell*" function in scanpy with counts_per_cell_after = 10,000; 2) top 1,000 highly variable genes were selected using the "*scanpy.api.pp.filter_genes_dispersion*" function in scanpy; 3) normalized gene expression for the selected top 1,000 highly variable genes was then transformed using log(1+x) transformation with natural logarithm; 4) the expression value is further standardized to a z-score within each batch based on specified batch ID (here, different sequencing technology is the batch ID) separately, and the standardized gene expression values were used as input for DESC. After the above filtering and data processing, there were 6,321 cells×1,000 highly variable genes remained in DESC analysis.

DESC analysis: We used two hidden layers for encoder with 64 nodes in the first hidden layer, and 32 nodes in the second hidden layer. Other parameters were set as default values. The final model is 1000-64-32-64-1000.

Remark: We also used this dataset to test the difference between Gaussian kernel and Student's t-distribution kernel in DESC. In the paper we used the Student's t-distribution as the kernel to measure the similarity between embedded point z_i for cell i and centroid μ_j for cluster j,

$$q_{ij} = \frac{\left(1+\|z_i-\mu_j\|^2/\alpha\right)^{-1}}{\sum_{j'}\left(1+\left\|z_i-\mu_{j'}\right\|^2/\alpha\right)^{-1}} \tag{1}$$

where $z_i = f_W(x_i) \in \mathbf{Z}$ corresponds to $x_i \in \mathbf{X}$ after embedding, α is the degree of freedom of the Student's t-distribution. But in order to evaluate whether the Student's t-distribution is appropriate, we also considered Gaussian-kernel, defined by

$$q_{ij} = \frac{exp\left(\frac{-\|z_i-\mu_j\|^2}{2\sigma^2}\right)}{\sum_{j'} exp\left(\frac{-\|z_i-\mu_j\|^2}{2\sigma^2}\right)} \tag{2}$$

as the clustering probability for each cell and keep all other parameters the same as before. We found the results based on Gaussian kernel are extremely unstable (**Supplementary Fig. 6**) and worse than those obtained from the Student's t-distribution.

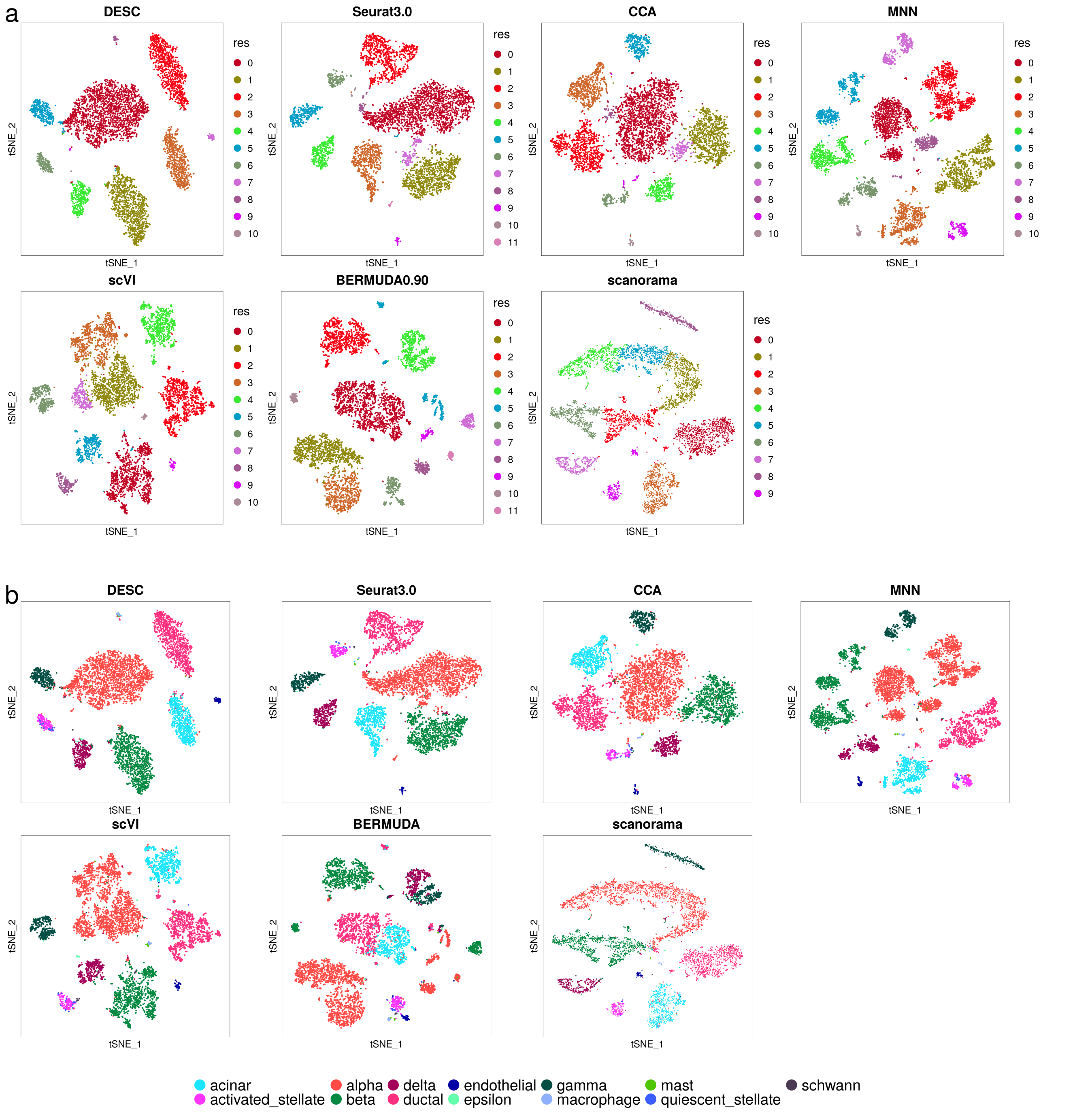

Supplementary Fig 4. Clustering results of the pancreatic islet dataset. **(a)** Color by cluster id obtained from 7 different batch removal methods. **(b)** Colored by the true cell type label (defined by the original paper).

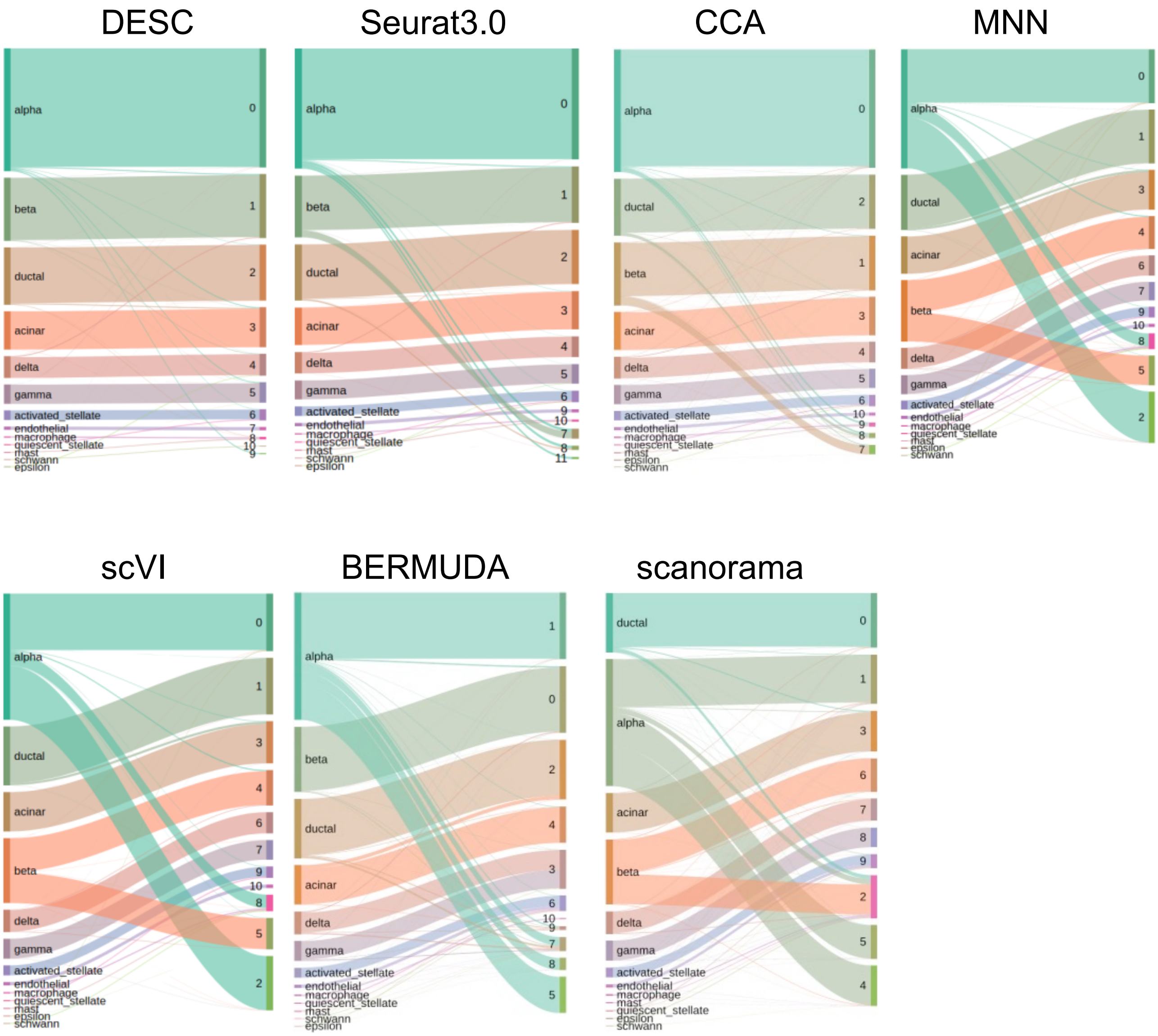

Supplementary Fig 5. The Sankey plots for 7 different methods. In addition, DESC yields accurate results for rare cell types such as schwann, mast, quiescent_stellate and macrophage (**Supplementary Fig. 4b**).

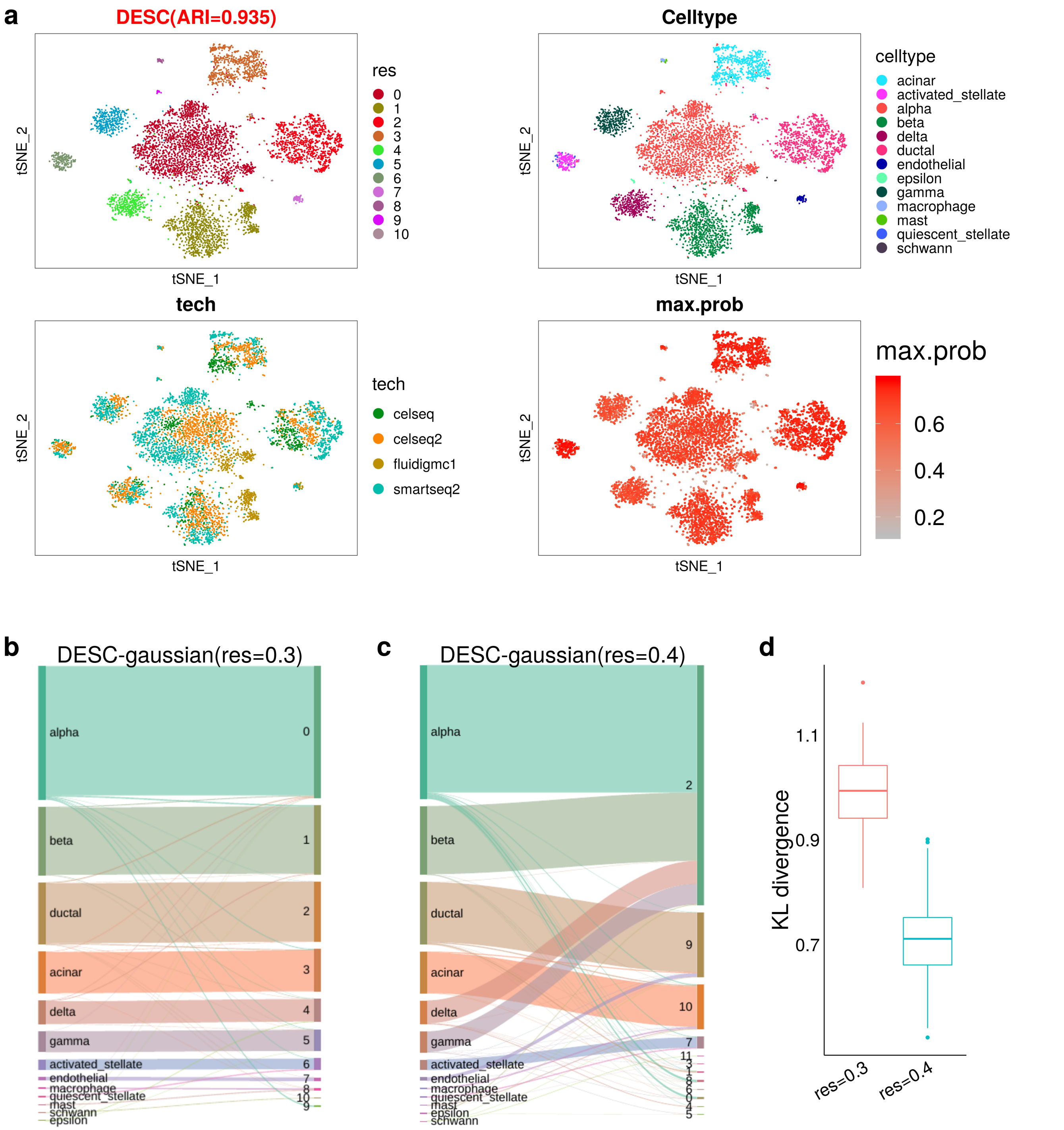

Supplementary Fig 6. The results of DESC for the pancreatic islet data with Gaussian kernel. **(a)** The results of DESC with Gaussian kernel when resolution=0.3. **(b)**The Sankey plot for clustering result when resolution=0.3. **(c)** The Sankey plot for clustering result when resolution=0.4. **(d)** The KL divergence of DESC clustering result. Compared with DESC using Student's t-distribution, the result of Gaussian kernel is not stable, with the ARIs being **0.935**, **0.409** when resolution=0.3, 0.4 respectively. However, for DESC with Student's t-distribution kernel, the ARIs are 0.9448, 0.9450, respectively, when resolution=0.3, 0.4. In addition, the median KL divergence is about 0.6 for Student's t-distribution kernel (**Fig. 4c**), but is 1.0 for Gaussian kernel. Due to these reasons, we chose to use the Student's t-distribution as the kernel.

Supplementary Note 4: analysis of the human PBMC data

This dataset was generated by Kang et al. (2018) Multiplexed droplet single-cell RNA-sequencing using natural genetic variation. Nature Biotechnology 36(1):89-94.

The data were downloaded from GEO (GSE96583), which include the raw gene count matrix, meta.data (t-SNE coordinates, ClusterID, celltype, and BatchID etc.) reported in the original paper. The downloaded data include 29,065 cells and 35,636 genes.

Cell filtering criteria: 1) eliminated cells that were labeled as multiplets and doublet.

Gene filtering criteria: 1) mitochondria genes were eliminated; 2) a gene was eliminated if the number of cells expressing this gene is <10.

Data processing: 1) gene expression levels for each cell was normalized using the "*scanpy.api.normalize_per_cell*" function in scanpy with counts_per_cell_after =10,000; 2) top 1,000 highly variable genes were selected using the "*scanpy.api.pp.filter_genes_dispersion*" function in scanpy; 3) normalized gene expression for the selected top 1,000 highly variable genes was then transformed using log(1+x) transformation with natural logarithm; 4) the expression is further standardized to a z-score transformation within each batch based on specified batch ID(here different conditions is the batch ID), and the standardized gene expression values were used as input for DESC. After the above filtering and data processing, there were 24,679 cells ×1,000 highly variable genes remained in DESC analysis.

DESC analysis: we used two hidden layers with 128 nodes in the first hidden layer, and 32 nodes in the second hidden layer. Other parameters were set default values. The final model was 1000-128-32-128-1000.

Remark: Due to memory issue, CCA and Seurat3.0 for this dataset were conducted on a CentOS Linux release 7.5.1804 (Core) with Intel(R) Xeon(R) CPU E7-4850 v4 @ 2.10GHz and total 1TB memory.

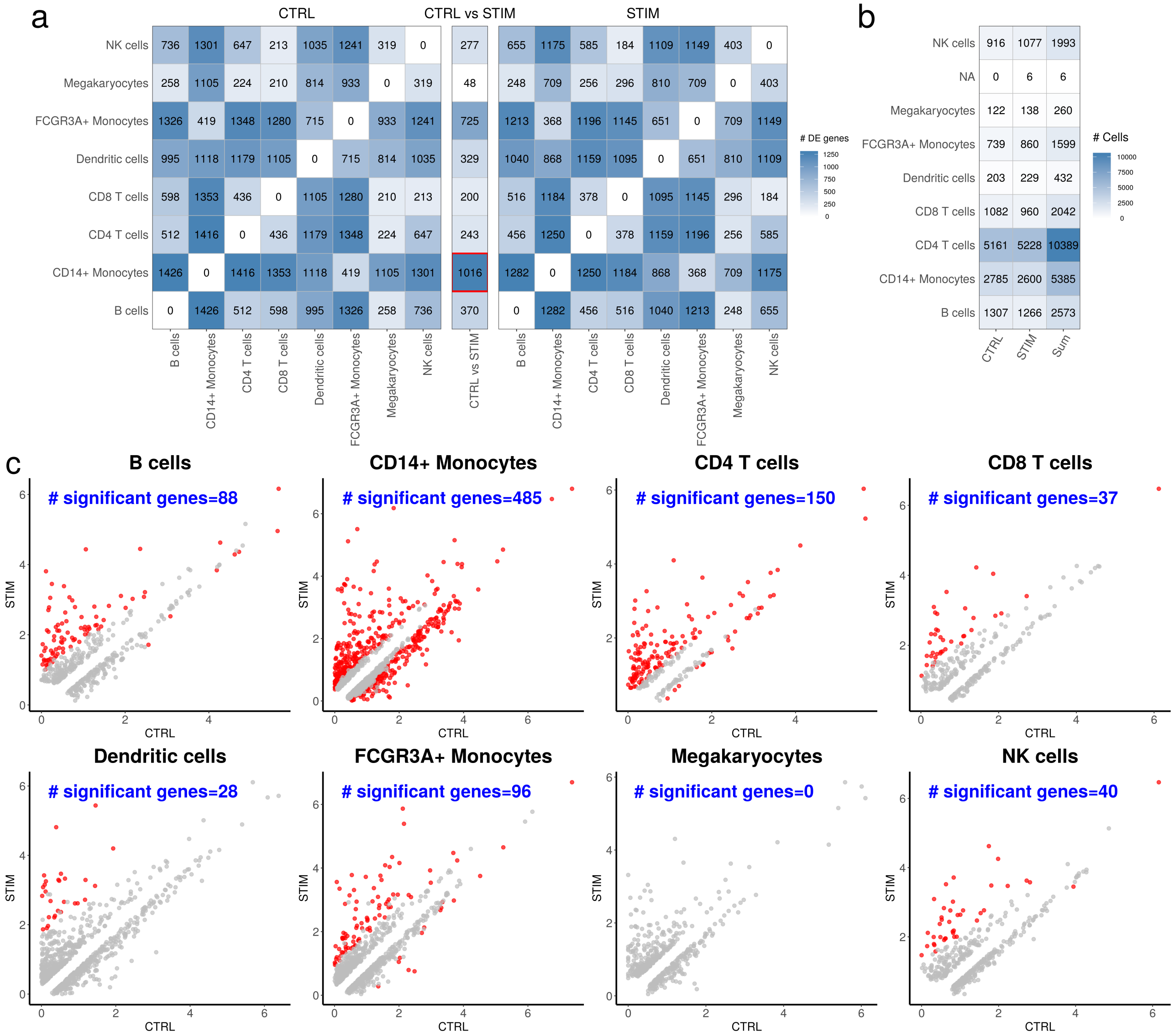

Supplementary Fig 7. Cell type labels were provided in the original paper (Kang et al. 2018). **(a)** Number of differentially expressed genes using Wilcoxon rank sum test with fold change > exp(0.25) and FDR adjusted p-value <0.01) between different cell types in the control group (left), the stimulated group (right), and differentially expressed genes between the control and the stimulated group within the same cell type (middle). **(b)** Number of cells in each cell type. **(c)** Comparison of gene expression levels between control and stimulated conditions on the PBMC data. Displayed are the average gene expressions across all cells in each condition for each cell type. Highlighted are differentially expressed genes using t-test with fold change > exp(0.25) and FDR adjusted p-value<10^{-50}. CD14+Monocytes have the largest number of differentially expressed genes between control and stimulated conditions.

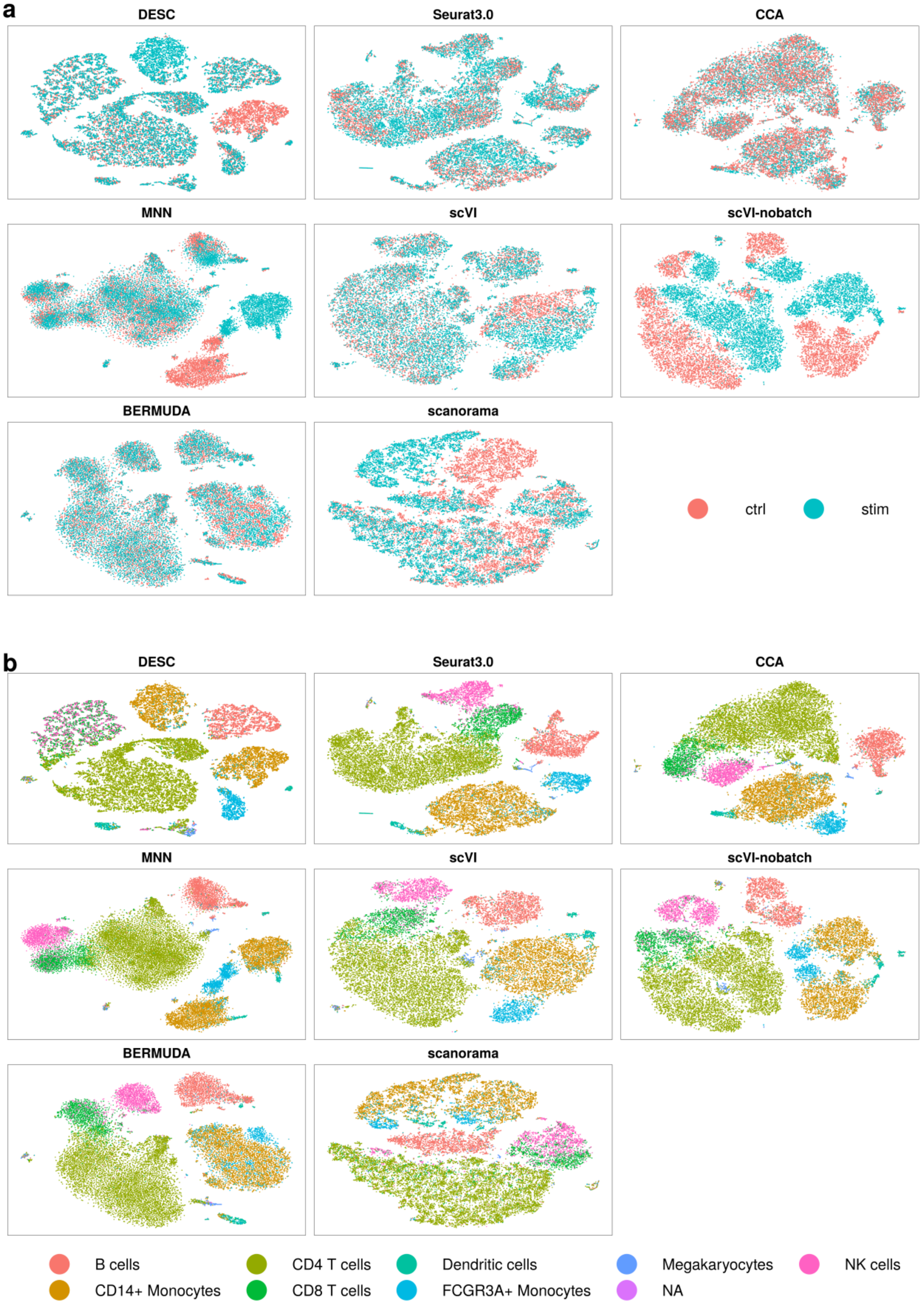

Supplementary Fig 8. The t-SNE plots of **DESC**, **Seurat3.0**, **CCA**, **MNN**, **scVI**, **scVI-nobatch**, **BERMUDA**, and **scanorama** for Kang et al (2018)'s dataset. **(a)** Cells were colored by BatchID; **(b)** Cells were colored by celltype. The method scVI-nobatch takes dataset as a whole without considering any batch information.

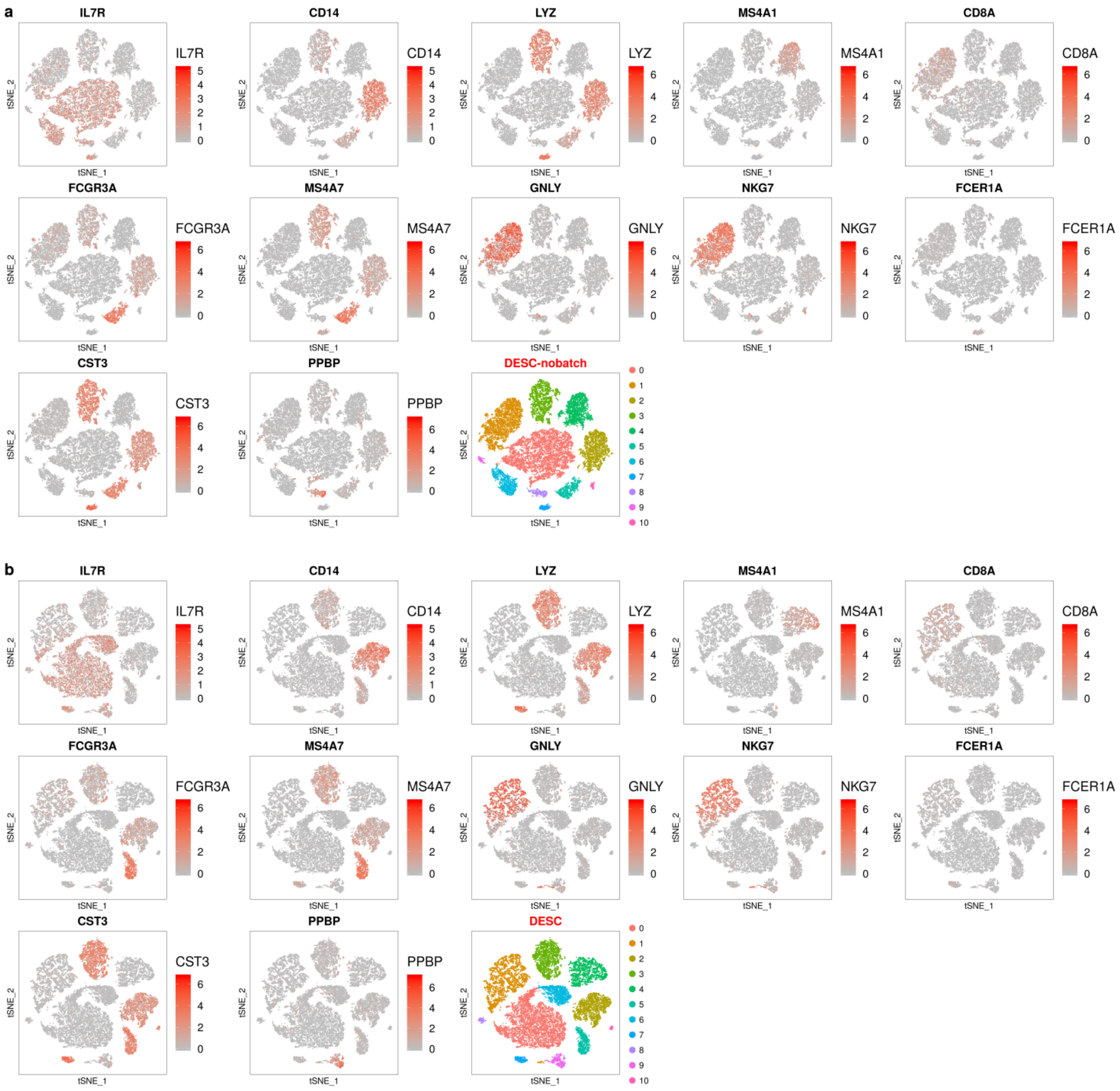

Supplementary Fig 9. Gene expression feature plots for cell-type specific marker genes and clustering results on the PBMC data for **(a) DESC** (resolution =0.6) and **(b) DESC nobatch** (resolution=0.6). IL7R (CD4 T cell marker), CD14 (CD14+ Monocyte marker), LYZ (CD14+ Monocyte marker), MS4A1 (B cell marker), CD8A (CD8 T cell marker), FCGR3A (FCGR3A+ monocyte marker), MS4A7 (FCGR3A+ Monocytes marker), GNLY (NK cell marker), NKG7 (NK cell marker), FCER1A (Dendritic Cell marker), CST3 (Dendritic Cell marker), PPBP (Megakaryocytes marker). **DESC nobatch** means no batch information was used in analysis.

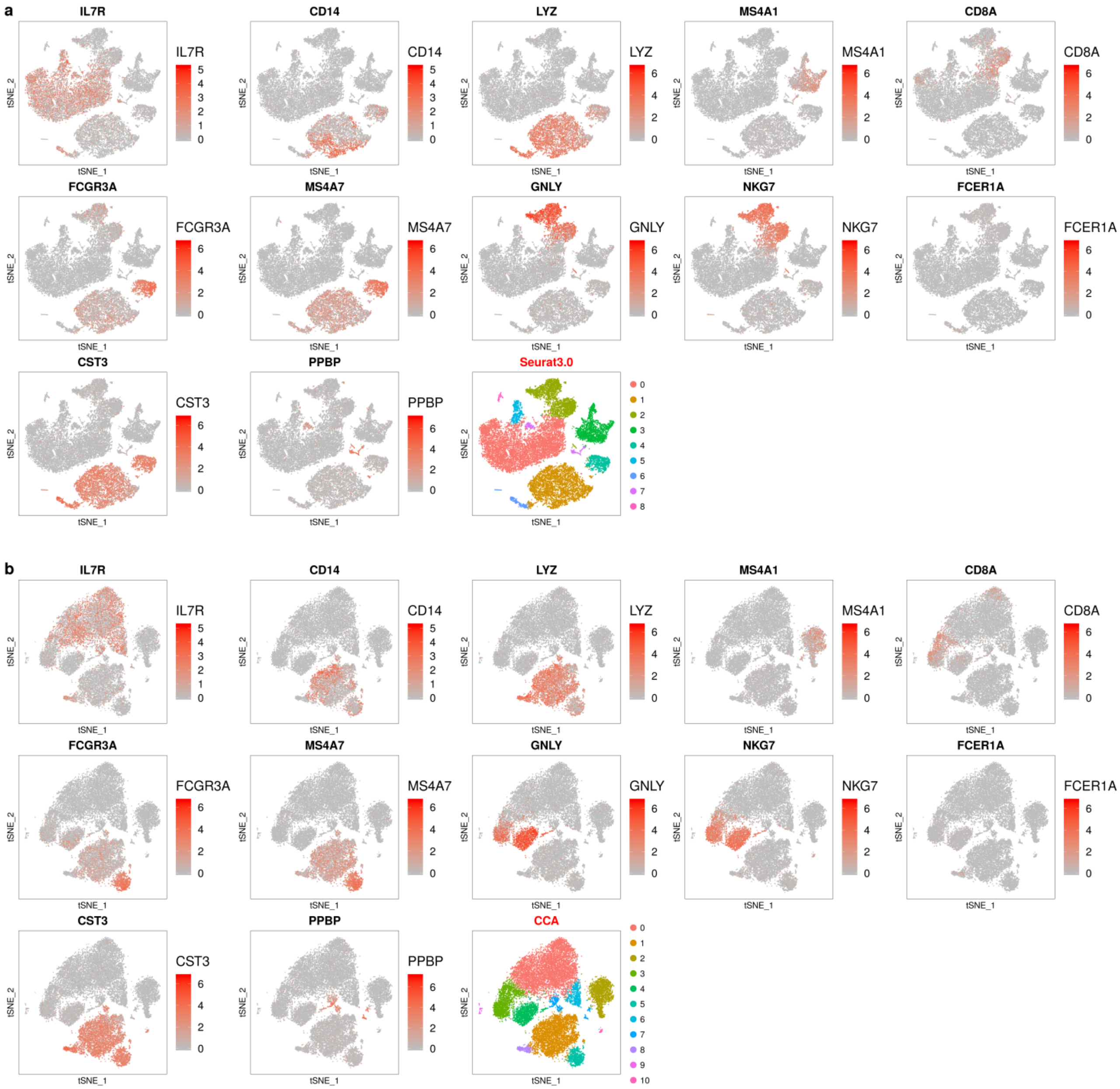

Supplementary Fig 10. Gene expression feature plots for cell-type specific marker genes and clustering results on the PBMC data for **(a) Seurat3.0** (resolution =0.1) and **(b) CCA** (resolution=0.2). IL7R (CD4 T cell marker), CD14 (CD14+ Monocyte marker), LYZ (CD14+ Monocyte marker), MS4A1 (B cell marker), CD8A (CD8 T cell marker), FCGR3A (FCGR3A+ monocyte marker), MS4A7 (FCGR3A+ Monocytes marker), GNLY (NK cell marker), NKG7 (NK cell marker), FCER1A (Dendritic Cell marker), CST3 (Dendritic Cell marker), PPBP (Megakaryocytes marker).

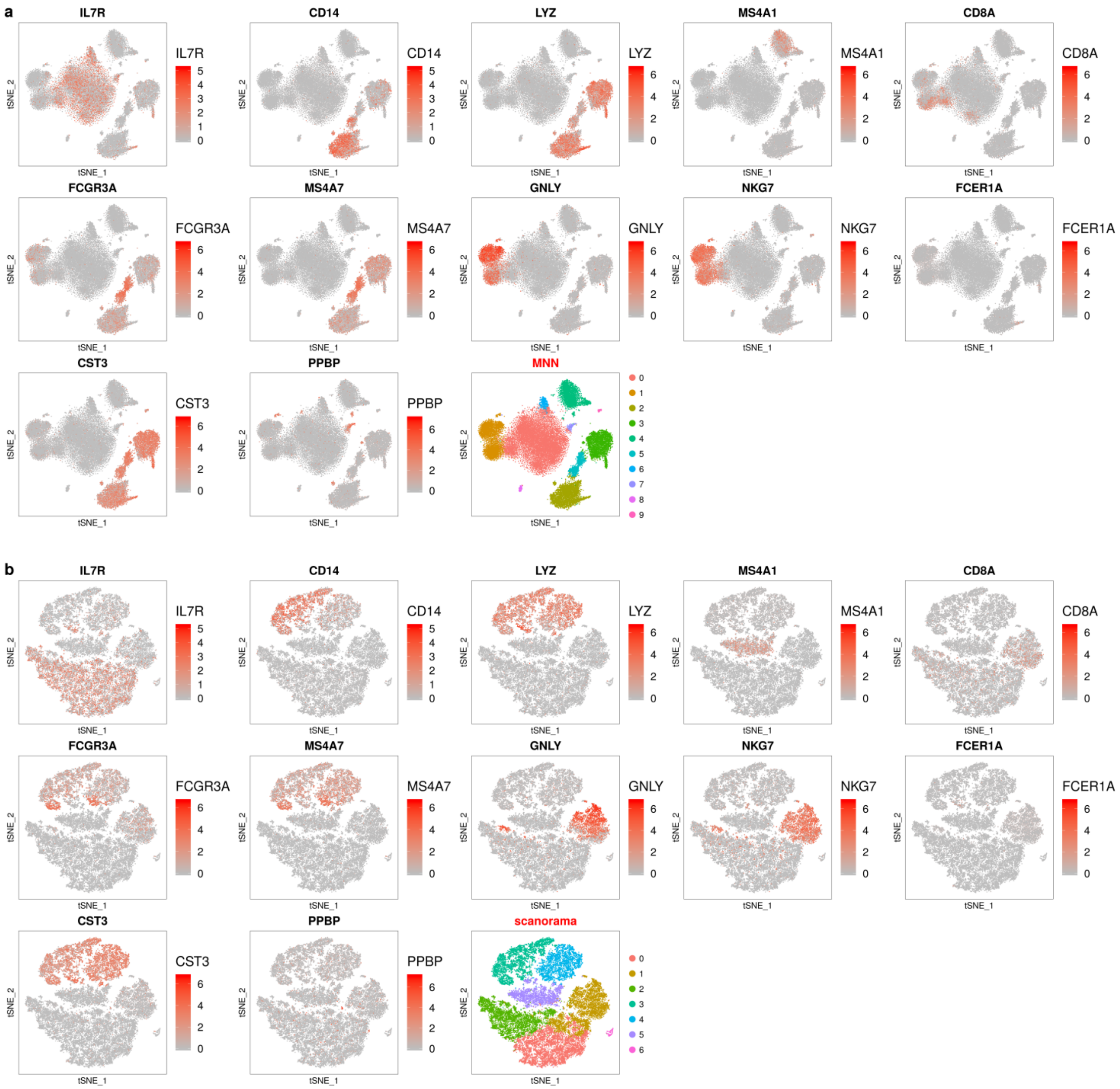

Supplementary Fig 11. Gene expression feature plots for cell-type specific marker genes and clustering results on the PBMC data for **(a) MNN** (resolution =0.3) and **(b) scanorama** (resolution=0.4). IL7R (CD4 T cell marker), CD14 (CD14+ Monocyte marker), LYZ (CD14+ Monocyte marker), MS4A1 (B cell marker), CD8A (CD8 T cell marker), FCGR3A (FCGR3A+ monocyte marker), MS4A7 (FCGR3A+ Monocytes marker), GNLY (NK cell marker), NKG7 (NK cell marker), FCER1A (Dendritic Cell marker), CST3 (Dendritic Cell marker), PPBP (Megakaryocytes marker).

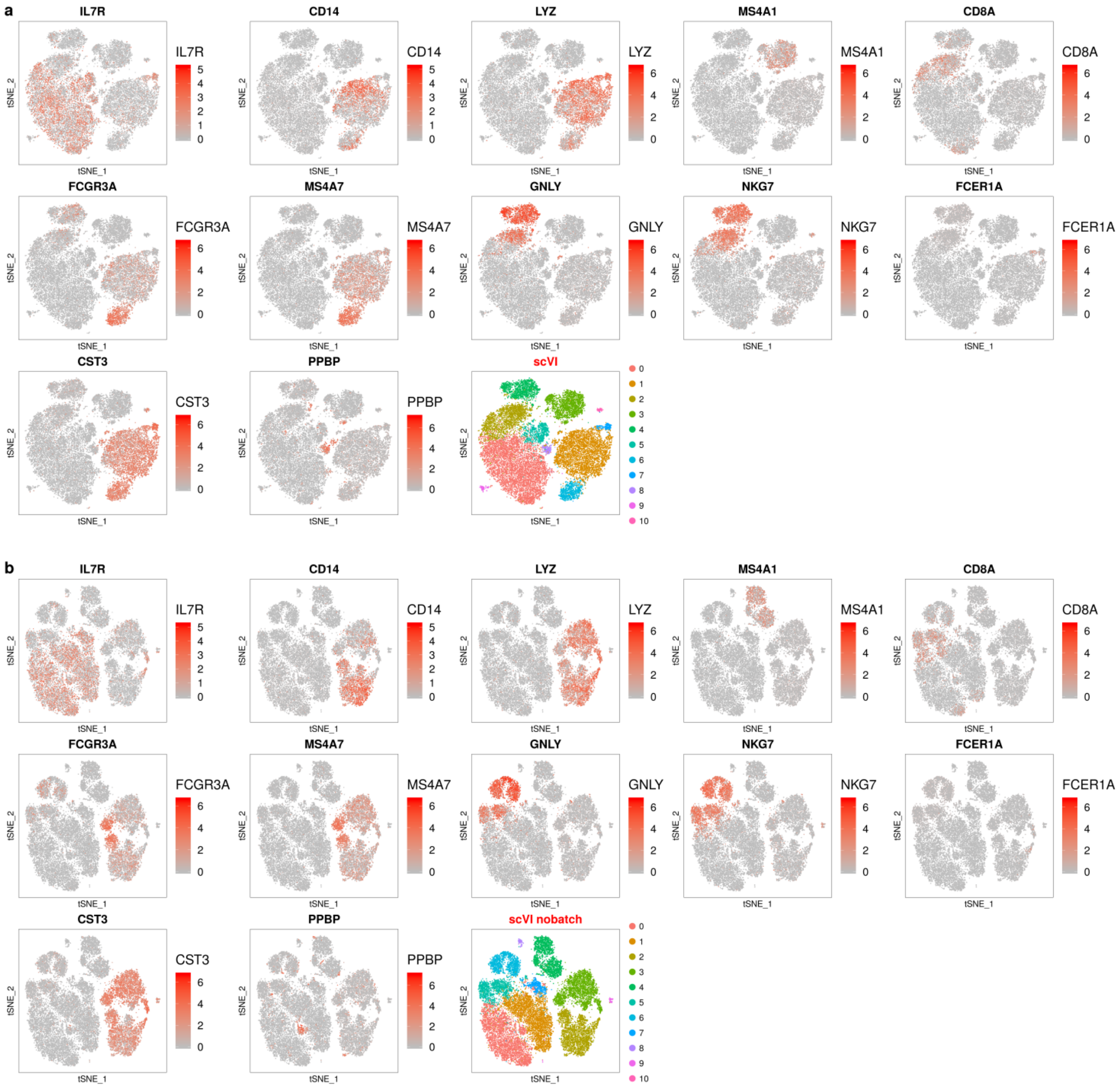

Supplementary Fig 12. Gene expression feature plots for cell-type specific marker genes and clustering results on the PBMC data for **(a) scVI** (resolution =0.4) and **(b) scVI nobatch** (resolution=0.4). IL7R (CD4 T cell marker), CD14 (CD14+ Monocyte marker), LYZ (CD14+ Monocyte marker), MS4A1 (B cell marker), CD8A (CD8 T cell marker), FCGR3A (FCGR3A+ monocyte marker), MS4A7 (FCGR3A+ Monocytes marker), GNLY (NK cell marker), NKG7 (NK cell marker), FCER1A (Dendritic Cell marker), CST3 (Dendritic Cell marker), PPBP (Megakaryocytes marker). **scVI nobatch** means no batch information was used in analysis.

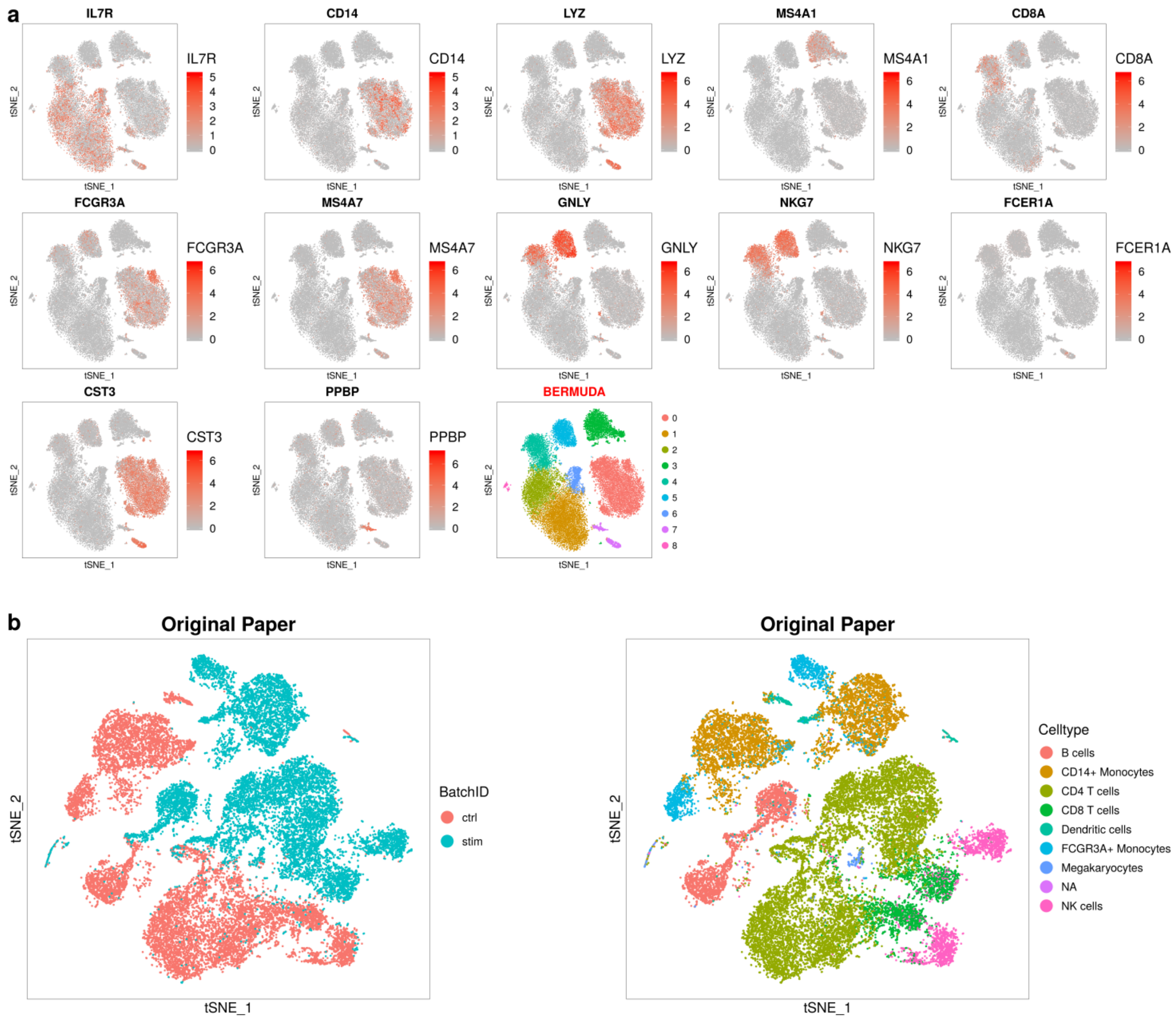

Supplementary Fig 13. Gene expression feature plots for cell-type specific marker genes and clustering results on the PBMC data for **(a) BERMUDA** (resolution =0.4). IL7R (CD4 T cell marker), CD14 (CD14+ Monocyte marker), LYZ (CD14+ Monocyte marker), MS4A1 (B cell marker), CD8A (CD8 T cell marker), FCGR3A (FCGR3A+ monocyte marker), MS4A7 (FCGR3A+ Monocytes marker), GNLY (NK cell marker), NKG7 (NK cell marker), FCER1A (Dendritic Cell marker), CST3 (Dendritic Cell marker), PPBP (Megakaryocytes marker); **(b)** The t-SNE plots reproduced based on t-SNE coordinates obtained from the original paper, the left panel is colored by batch and the right panel is colored by cell type.

Supplementary Note 5: Analysis of the mouse bone marrow data

This dataset was generated by Paul et al. (2015). Transcriptional Heterogeneity and Lineage Commitment in Myeloid Progenitors. Cell 163, 1663-167.

The raw gene expression data, which include count matrix, meta.data (t-SNE coordinates, ClusterID, celltype, and BatchID) were download from GSE72857. Here we simply downloaded this data using command "*scanpy.datasets.paul15()"* in the scanpy python software. The downloaded data include 2,730 cells and 8,716 genes.

Cell filtering criteria: 1) we did not filter out any cells because the downloaded data were already prefiltered.

Gene filtering criteria: 1) mitochondrial genes were eliminated; 2) a gene was eliminated if the number of cells expressing this gene is <10.

Data processing: 1) gene expression levels for each cell was normalized using the "*scanpy.api.normalize_per_cell*" function in scanpy with counts_per_cell_after =10,000; 2) top 1,000 highly variable genes were selected using the "*scanpy.api.pp.filter_genes_dispersion*" function in scanpy; 3) normalized gene expression for the selected top 1,000 highly variable genes was then transformed using log(1+x) transformation with natural logarithm; 4) the expression value is further standardized to a z-score across all cells, and the standardized gene expression values were used as input for DESC. After the above filtering and data processing, there were 2,730 cells×1,000 highly variable genes remained in DESC analysis.

DESC analysis: We used two hidden layers for encoder with 64 nodes in the first hidden layer, and 32 nodes in the second hidden layer. The final model is 1000-64-32-64-1000. Since the number of cells is relatively small, we use tol=0.005 for this data. Other parameters were set as default values. For scVI, we used the top 2,000 highly variable genes as usual.

Supplementary Note 6: Analysis of the human monocyte data

This dataset was generated by our group, which can be downloaded from GEO (accession number GSE146974). This dataset was generated from human peripheral blood mononuclear clear cells by Ficoll Separation followed by CD14 and CD16 positive cell selection. Since the CD14 and CD16 antibodies are not 100% specific, some T cells were also present in the scRNA-seq data. We performed clustering analysis using Louvain's algorithm for each batch and identified 288 T cells in total based on the T cell marker genes CD3D, CD3E and CD3G. After removing these 288 T cells, there are 10,878 cells and 21,289 genes, which was processed and sequenced at three different days, resulting in three batches (3,640 cells in T1, 4,833 cells in T2 and 2,405 cells in T3) left in the remaining analysis. **Figs. 7- 8** and **Supplementary Figs. 14 and 15** show the results for analysis of these data.

Human monocyte preparation: Monocyte preparation uses a modification of published protocols. Briefly, ~20 ml blood drawn in sodium heparin was processed immediately in the lab in the Clinical Research Center at Columbia University. PBMCs were isolated by gradient Ficoll paque centrifugation, which maintains cell viability and prevents ex vivo activation during cell recovery. Cells were stained with antibodies against human HLADR, CD14 and CD16 and monocyte subsets defined as HLADR+CD14++CD16-(classical), HLADR+CD14++CD16+ (intermediate), HLADR+CD14dim/CD16++ (nonclassical, patrolling monocyte). DAPI staining was used to exclude dead cells. Monocytes were sorted by a BD Influx Sorter into tubes for real-time 10x Genomics analysis.

Cell filtering criteria: 1) eliminated cells with percentage of mitochondrial UMI counts >25%; 2) eliminated cells with gene counts <200; 3) eliminated cells with total UMI counts <1000;

Gene filtering criteria: 1) mitochondrial genes were eliminated; 2) a gene was eliminated if the number of cells expressing this gene is <10.

Data processing: 1) gene expression levels for each cell was normalized using the "*scanpy.api.normalize_per_cell*" function in scanpy with counts_per_cell_after = 10,000 ; 2) top 1,000 highly variable genes were selected using the "*scanpy.api.pp.filter_genes_dispersion*" function in scanpy; 3) normalized gene expression for the selected top 1,000 highly variable genes was then transformed using log(1+x) transformation with natural logarithm; 4) the expression value was further standardized to a z-score transformation for cells within each batch based on specified batch ID, and the standardized gene expression values were used as input for DESC.

After the above filtering and data processing, there were 10,878 cells×1,000 highly variable genes remained in DESC analysis.

DESC analysis: We used two hidden layers for encoder with 128 nodes in the first hidden layer, and 32 nodes in the second hidden layer. We use *tol*=0.005 and *resolution*=0.7 for this data. Other parameters were set as default values. The final model is 1000-128-32-128-1000.

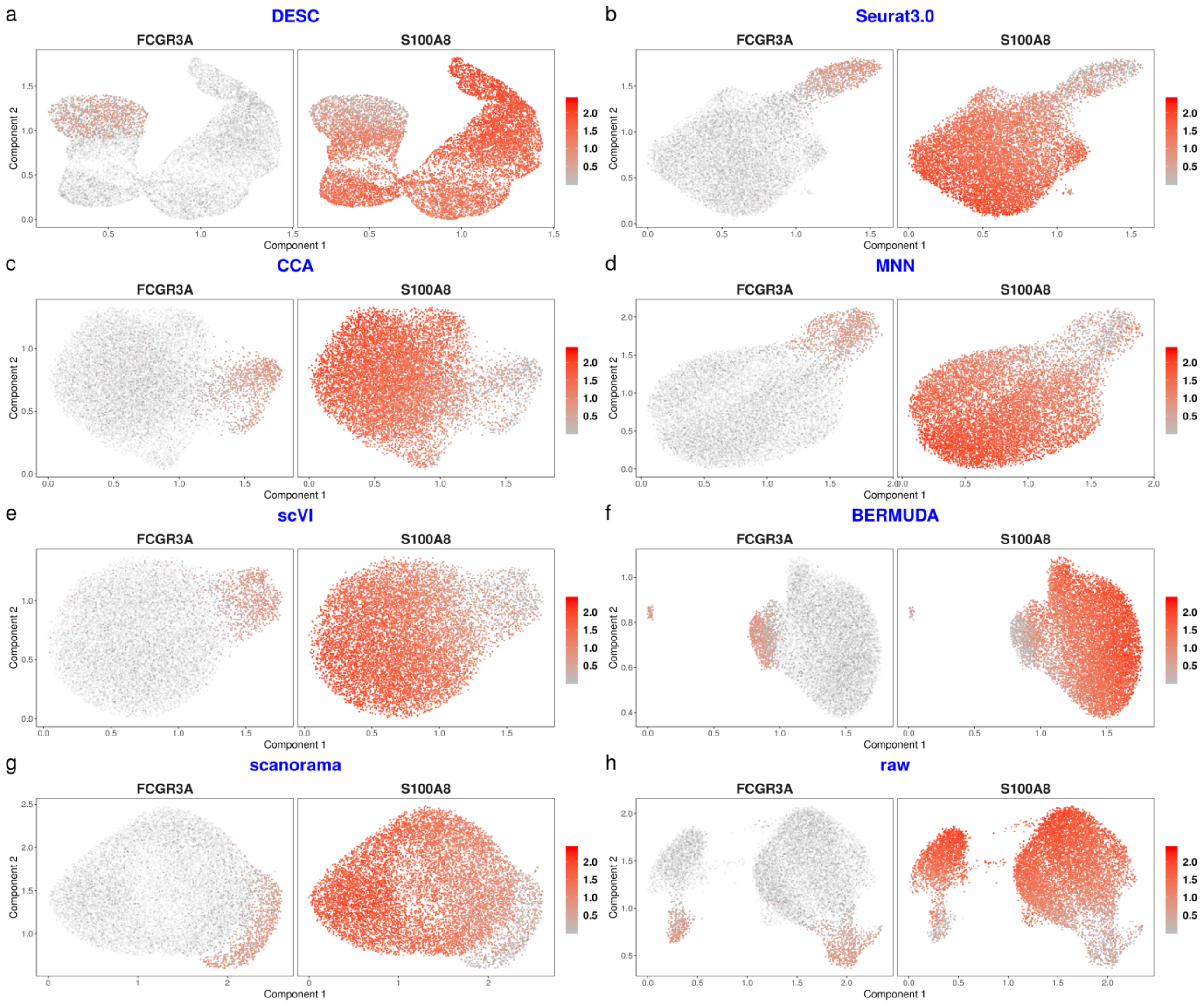

Supplement Fig 14. The gene expression feature plots for marker genes FCGR3A (non-classical monocyte) and S100A8 (classical monocyte) for different methods. **(a)** Gene expression feature plots based on UMAP using low-dimensional representation from DESC as input; **(b)** Gene expression feature plots based on UMAP using pca components obtained from Seurat3.0 as input; **(c)** Gene expression feature plots based on UMAP using cca components obtained from method CCA as input; **(d)** Gene expression feature plots based on UMAP using pca components of corrected gene expression values from method MNN as input; **(e)** Gene expression feature plots based on UMAP using representation (bottleneck layer) obtained from scVI as input; **(f)** Gene expression feature plots based on UMAP using representation (bottleneck layer) obtained from BERMUDA as input; **(g)** Gene expression feature plots based on UMAP using representation obtained from scanorama as input; **(h)** Gene expression feature plots based on UMAP using the raw gene expression matrix as input.

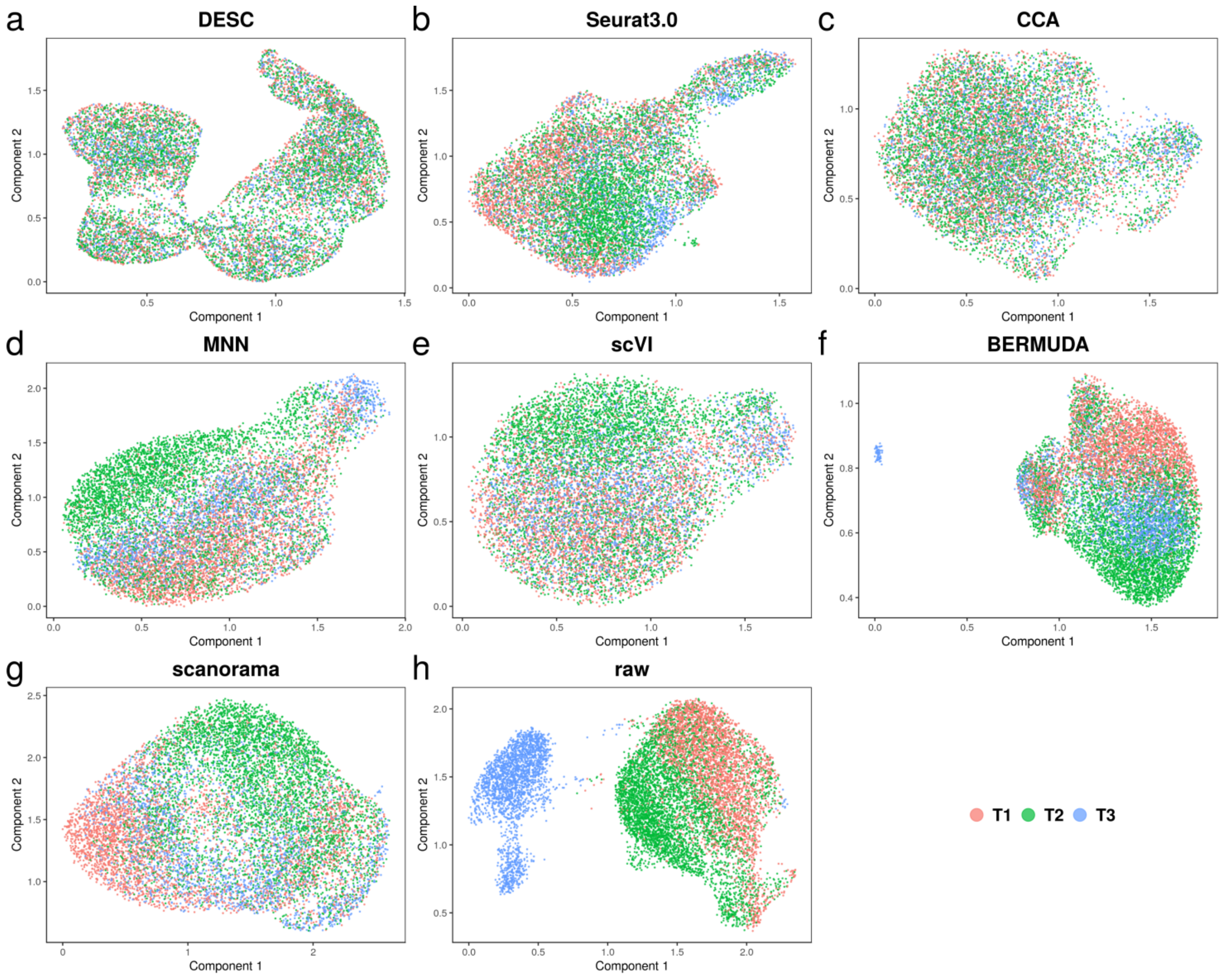

Supplement Fig 15. The batch distribution plots for different methods. (a) batch distribution based on UMAP using low-dimensional representation from DESC as input; (b) batch distribution based on UMAP using pca components obtained from Seurat3.0 as input; (c) batch distribution based on UMAP using cca components obtained from method CCA as input; (d) batch distribution based on UMAP using pca components of corrected gene expression values from method MNN as input; (e) batch distribution based on UMAP using representation (bottleneck layer) obtained from scVI as input; (f) batch distribution based on UMAP using representation (bottleneck layer) obtained from BERMUDA as input; (g) batch distribution based on UMAP using representation obtained from scanorama as input; (h) batch distribution based on UMAP using the raw gene expression matrix as input.

If a method is effective in removing technical batch effect, the estimated pseudotimes across three batches should be similar. Therefore, we used Kolmogorov-Smirnov test to examine the difference of pseudotime distributions among different batches. **Supplementary Table 5** shows that results obtained from DESC have the smallest distributional differences, providing additional evidence that DESC performs the best in batch effect removal.

Supplementary Table 5. P-values for comparing the pseudo-time distributions among the three batches using Kolmogorov-Smirnov test.

Method	T1 v.s. T2	T1 v.s. T3	T2 v.s. T3
raw+monocle3	< 2.2e-16	< 2.2e-16	< 2.2e-16
DESC+monocle3	4.051e-4	1.443e-08	2.169e-2
Seurat3.0+monocle3	< 2.2e-16	< 2.2e-16	4.666e-9
CCA+monocle3	< 2.2e-16	< 2.2e-16	< 2.2e-16
MNN+monocle3	3.864e-16	< 2.2e-16	< 2.2e-16
scVI+monocle3	< 2.2e-16	< 2.2e-16	< 2.2e-16
BERMUDA+monocle3	< 2.2e-16	< 2.2e-16	< 2.2e-16
scanorama+monocle3	< 2.2e-16	< 2.2e-16	< 2.2e-16

It can be seen that DESC has the largest p values, indicating that the differences between T1, T2 and T3 are the smallest compared to other methods.

Supplementary Note 7: Analysis of the mouse brain data with 1.3 million cells

The data were downloaded from 10X website https://support.10xgenomics.com/single-cell-gene-expression/datasets/1.3.0/1M_neurons. The original data include 1,306,127 cells and 27,998 genes.

Cell filtering criteria: 1) eliminated cells with gene counts <200.

Gene filtering criteria: 1) a gene was eliminated if the number of cells expressing this gene is <20.

Data processing: 1) gene expression levels for each cell was normalized using the "*scanpy.api.normalize_per_cell*" function in scanpy with counts_per_cell_after =10,000; 2) top 1,000 highly variable genes were selected using the "*scanpy.api.pp.filter_genes_dispersion*" function in scanpy; 3) normalized gene expression for the selected top 1,000 highly variable genes was then transformed using log(1+x) transformation with natural logarithm; 4) the expression is further standardized to a z-score, and the standardized gene expression values were used as input for DESC. After the above filtering and data processing, there are 1,292,537cells ×1,000 highly variable genes remained in DESC.

DESC analysis: we used two hidden layers with 64 nodes in the first hidden layer, and 32 nodes in the second hidden layer. The parameters we used were n_neighbors=15, batch_size=20000, tol=0.008, louvain_resolution=0.2, use_GPU=True, is_stacked=False, pretrain_epochs=10, epochs_fit=2. Other parameters were set to default values. The final model is 1000-64-32-64-1000.

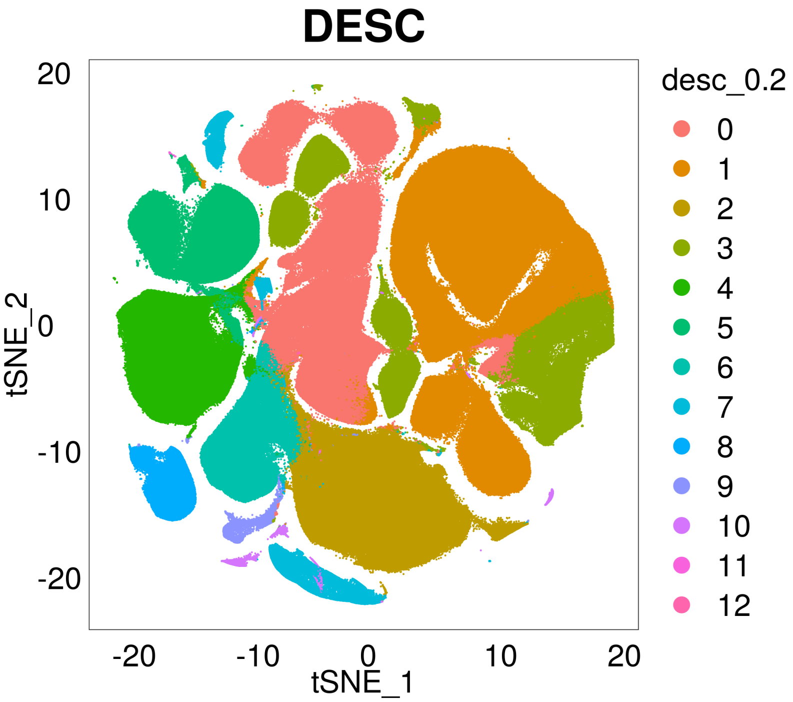

Supplementary Fig 16. Clustering result of DESC on the 1.3 million mouse data.

In order to compare computing time and memory usage for three popular clustering methods and different numbers of cells, we randomly selected 1,000, 2,000, 5,000, 10,000, 20,000, 40,000, 50,000, 100,000, 300,000, 500,000, 1,000,000, 1,300,000 cells from the above 1.3 million cell dataset. **Note:** The inputs of all methods are the top 1000 genes selected by function '*filter_genes_dispersion*' in python

module *scanpy*. The number of epochs for both DESC and scVI was set to 100, and for Seurat3.0 we used their default parameters.

For each method, we recorded its memory usage every second when the method was running. The memory we reported is the maximal memory use during the running of the corresponding method. For **DESC**, **DESC_GPU, DESC_multicpu and Seurat3.0** (Seurat with version 3.0.0) method, we successfully completed analyses for all datasets. Due to the huge computational cost of scVI, we only run analysis with less than 500,000 cells for **scVI, scVI_multicpu and scVI_GPU**. Note that the running time of scVI with single CPU for 1,000,000 cells exceeds 200,000 seconds. Even with a GPU, the running time of scVI is still longer than 150,000 seconds. For 1.3 million cells, DESC with a single GPU can finish the clustering analysis within 3.5 hours and only takes less than 10GB memory. In contrast, Seurat3.0 requires more than 60GB memory to analyze 1.3 million cells, which is not feasible for most personal computers. Although scVI also can utilize GPU, it is extremely time-consuming.

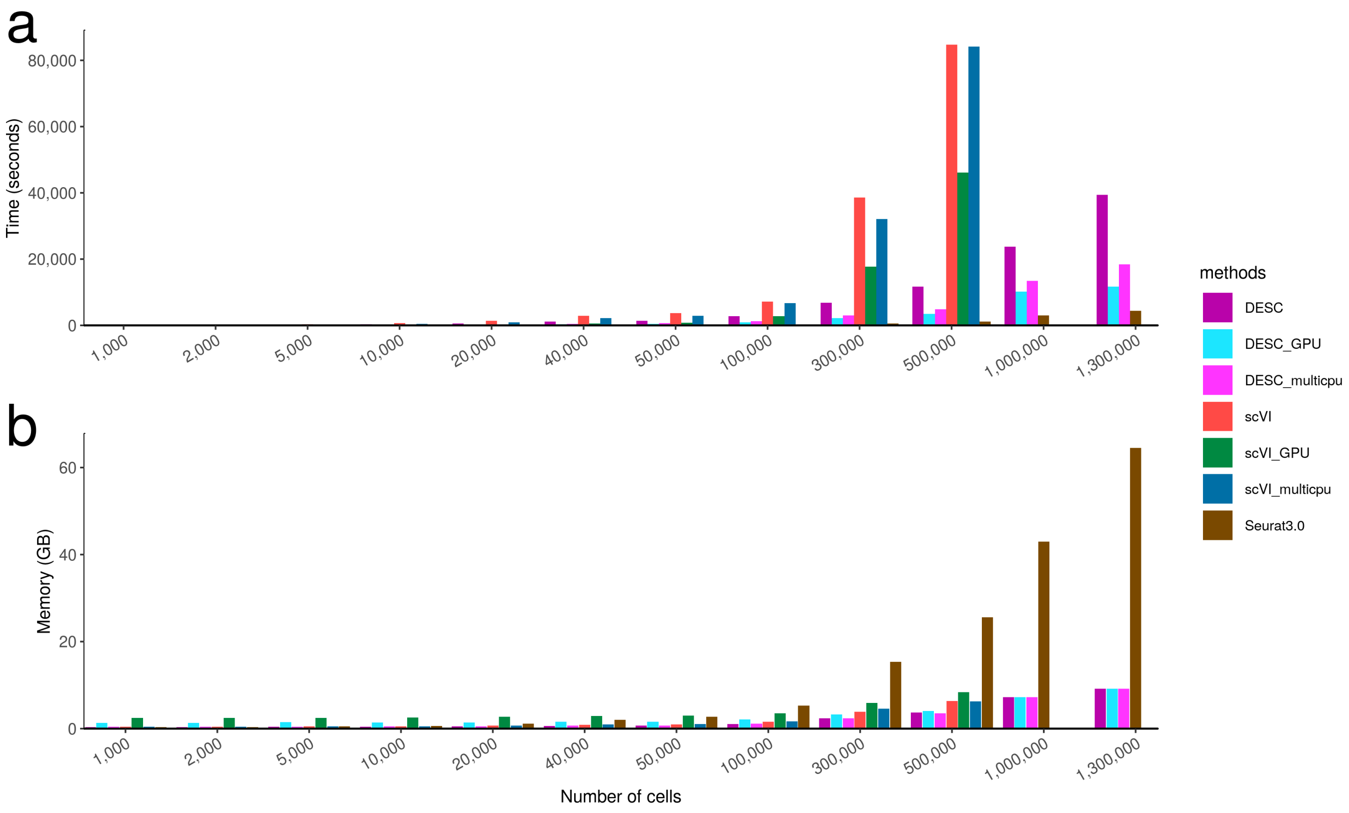

Supplementary Fig 17. Comparison of running time **(a)** and memory usage **(b)** of three clustering method for datasets with various numbers of cells, which were randomly sampled from the 1.3 million mouse brain dataset. DESC: used a single CPU; DESC_GPU: used a single GPU; DESC_multicpu: used 10 CPUs; ScVI: used a single CPU. scVI_GPU: used a single GPU; scVI_multicpu: used 10 CPUs. **DESC** used *desc.train* function in python module *desc with version 1.0.0.5*. **Seurat** used *FindClusters* function in R package Seurat version 2.3.4. **Seurat3.0** used *FindClusters* function in R package Seurat version 3.0.0. **scVI** used *UnsupervisedTrainer* function in python module scvi version 0.3.0. **Remark**: we analyzed this dataset on Ubuntu 16.04.4 LTS with Intel(R) Xeon(R) CPU E5-2620 v4 @ 2.10GHz and 128GB memory.

All data analyses reported in this paper were conducted on Ubuntu 18.04.1 LTS with Intel® Core (TM) i7-8700K CPU @ 3.70GHz and 64GB memory, except for the 1.3 million cells mouse brain data. For

the 1.3 million cells mouse brain dataset, we analyzed on Ubuntu 16.04.4 LTS with Intel(R) Xeon(R) CPU E5-2620 v4 @ 2.10GHz and total 128GB memory.

Supplementary References

[1] Blondel VD, Guillaume JL, Lambiotte R, Lefebvre E. Fast unfolding of communities in large networks, Journal of Statistical Mechanics: Theory and Experiment 2008, 2008.

[2] Butler A, Hoffman P, Smibert P, Papalexi E, Satija R. Integrating single-cell transcriptomic data across different conditions, technologies, and species. Nature Biotechnology, 36:411-420 (2018).

[3] Grün D, Muraro MJ, Boisset JC, Wiebrands K et al. De novo prediction of stem cell identity using single-cell transcriptome data. Cell Stem Cell, 19:266-277 (2016).

[4] Haghverdi L, Lun AT, Morgan MD, Marioni JC. Batch effects in single-cell RNA-sequencing data are corrected by matching mutual nearest neighbors. Nature Biotechnology, 36: 421-427 (2018).

[5] Hie, B., Bryson, B. & Berger, B. Efficient integration of heterogeneous single-cell transcriptomes using Scanorama. *Nature Biotechnology* **37**, 685 (2019).

[6] Hyun MK, Subramaniam M, Targ S, Nguyen M, Maliskova L, McCarthy E, Wan E, Wong S, Byrnes L, Lanata CM, Gate RE, Mostafavi S, Marson A, Zaitlen N, Criswell LA, Ye CJ. Multiplexed droplet single-cell RNA-sequencing using natural genetic variation. Nature Biotechnology, 36:89-94 (2018).

[7] Lawlor N, George J, Bolisetty M, Kursawe R et al. Single-cell transcriptomes identify human islet cell signatures and reveal cell-type-specific expression changes in type 2 diabetes. Genome Research, 27: 208-222 (2017).

[8] Lopez R, Regier J, Cole M, Jordan MI, Yosef N. Deep generative modeling for single-cell transcriptomics. Nature Methods, 15:1053-1058 (2018).

[9] Muraro MJ, Dharmadhikari G, Grün D, Groen N et al. A single-cell transcriptome atlas of the human pancreas. Cell Systems, 3:385-394 (2016).

[10] Paul F, Arkin Y, Giladi A, Jaitin DA et al. Transcriptional heterogeneity and lineage commitment in myeloid progenitors. Cell, 163:1663-167 (2015).

[11] Peng YR, Shekhar K, Yan W, Herrmann D, Sappington A, Bryman GS, van Zyl T, Do MTH, Regev A, Sanes JR. Molecular classification and comparative taxonomics of foveal and peripheral cells in primate retina. Cell, 176:1222-1237 (2019).

[12] Segerstolpe A, Palasantza A, Eliasson P, et al. Single-cell transcriptome profiling of human pancreatic islets in health and type 2 diabetes. Cell Metabolism, 24:593–607 (2016).

[13] Wang, T. *et al.* BERMUDA: a novel deep transfer learning method for single-cell RNA sequencing batch correction reveals hidden high-resolution cellular subtypes. *Genome Biology* **20**, 165 (2019).
