# Supplementary material for: Deep learning enables accurate clustering with batch effect removal in single-cell RNA-seq analysis
Source: Nat Commun. 2020 May 11;11:2338. doi: 10.1038/s41467-020-15851-3 (PMC7214470; doi:10.1038/s41467-020-15851-3)
Supplement: Supplementary file 3 — Reporting Summary [file 41467_2020_15851_MOESM3_ESM.pdf]

nature research

Corresponding author(s): Gang Hu, Mingyao Li

Last updated by author(s): Mar 18, 2020

Reporting Summary

Nature Research wishes to improve the reproducibility of the work that we publish. This form provides structure for consistency and transparency in reporting. For further information on Nature Research policies, see Authors & Referees and the Editorial Policy Checklist.

Statistics

For all statistical analyses, confirm that the following items are present in the figure legend, table legend, main text, or Methods section.

n/a	Confirmed	
☐	☒	The exact sample size (*n*) for each experimental group/condition, given as a discrete number and unit of measurement
☐	☒	A statement on whether measurements were taken from distinct samples or whether the same sample was measured repeatedly
☐	☒	The statistical test(s) used AND whether they are one- or two-sided<br>*Only common tests should be described solely by name; describe more complex techniques in the Methods section.*
☒	☐	A description of all covariates tested
☒	☐	A description of any assumptions or corrections, such as tests of normality and adjustment for multiple comparisons
☒	☐	A full description of the statistical parameters including central tendency (e.g. means) or other basic estimates (e.g. regression coefficient) AND variation (e.g. standard deviation) or associated estimates of uncertainty (e.g. confidence intervals)
☐	☒	For null hypothesis testing, the test statistic (e.g. *F*, *t*, *r*) with confidence intervals, effect sizes, degrees of freedom and *P* value noted<br>*Give P values as exact values whenever suitable.*
☒	☐	For Bayesian analysis, information on the choice of priors and Markov chain Monte Carlo settings
☒	☐	For hierarchical and complex designs, identification of the appropriate level for tests and full reporting of outcomes
☒	☐	Estimates of effect sizes (e.g. Cohen's *d*, Pearson's *r*), indicating how they were calculated

Our web collection on statistics for biologists contains articles on many of the points above.

Software and code

Policy information about availability of computer code

Data collection	No software was used for data collection.
Data analysis	python modules:<br>DESC (https://github.com/eleozzr/desc), MNN (1.3.6), scanpy (1.3.6), scanorama (1.4), keras (2.1.5), tensorflow (1.7.0), scvi (0.3.0),<br>R packages:<br>Seurat (v2.3.4), Seurat(v3.0.0), Monocle3 (alpha), googleVis (v0.6.4)<br>Python + R: BERMUDA (https://github.com/txWang/BERMUDA)

For manuscripts utilizing custom algorithms or software that are central to the research but not yet described in published literature, software must be made available to editors/reviewers. We strongly encourage code deposition in a community repository (e.g. GitHub). See the Nature Research guidelines for submitting code & software for further information.

Data

Policy information about availability of data

All manuscripts must include a data availability statement. This statement should provide the following information, where applicable:

- Accession codes, unique identifiers, or web links for publicly available datasets
- A list of figures that have associated raw data
- A description of any restrictions on data availability

The data sets used in this manuscript are available through the following web links:
(1) Bipolar cells from macaque retina (2019 Cell), GSE118480.
(2) Human pancreatic islet data, CelSeq (GSE81076), CelSeq2 (GSE85241), Fluidigm C1 (GSE86469), and SMART-Seq2 (E-MTAB-5061). The combined raw data matrix and associated metadata file can be downloaded from https://www.dropbox.com/s/1zxbn92y5du9pu0/ pancreas_v3_files.tar.gz?dl=1.
(3) Mouse hematopoietic stem cells with bone marrow (2015 Cell). The data can be downloaded from: https://www.ncbi.nlm.nih.gov/geo/download/?acc=GSE72857&format=file&file=GSE72857_umitab.txt.gz.

(4) Human PBMC data generated by Kang et al (2018 Nature Biotech): https://ftp.ncbi.nlm.nih.gov/geo/series/GSE96nnn/GSE96583/suppl/.
(5) The human monocyte data generated by us can be downloaded from: https://www.dropbox.com/s/kz5x8vskvvf7u8f/monocyte_desc_use.tar.gz?dl=0
(6) 1.3 million mouse data generated by 10x Genomics: https://support.10xgenomics.com/single-cell-gene-expression/datasets/1.3.0/1M_neurons.

Field-specific reporting

Please select the one below that is the best fit for your research. If you are not sure, read the appropriate sections before making your selection.

☒ Life sciences ☐ Behavioural & social sciences ☐ Ecological, evolutionary & environmental sciences

For a reference copy of the document with all sections, see nature.com/documents/nr-reporting-summary-flat.pdf

Life sciences study design

All studies must disclose on these points even when the disclosure is negative.

Sample size	1) Bipolar cells from macaque retina (2019 Cell): 30,302 cells; (2) Human pancreatic islet data: 6,321 cell;(3) Mouse hematopoietic stem cells with bone marrow (2015 Cell): 2,730 cells; (4) Human PBMC data generated by Kang et al (2018 Nature Biotech): 24,679 cells. (5) Human monocyte data generated by us: 11,166 cells; (6) Mouse Brain data by 10x Genomics: 1,306,127 cells.
Data exclusions	(1) Bipolar cells from macaque retina (2019 Cell): mitochondrial genes were eliminated and a gene was eliminated if the number cells expressing this gene is<10; (2) Human pancreatic islet data: no data were excluded. (3) Mouse hematopoietic stem cells with bone marrow (2015 Cell): no data were exclued; (4) Human PBMC by Kang et al (2018 Nature Biotech): we eliminated cells that were labeled as multiplets or doublets; (5) Human monocyte data generated by us: we eliminated bad quality cells (percentage of mitochondrial UMI counts >25% or UMI counts <1000 or gene counts <200). (7) Mouse Brain data by 10x Genomics: we eliminated cells with gene counts <200.
Replication	NA.
Randomization	NA.
Blinding	NA.

Reporting for specific materials, systems and methods

We require information from authors about some types of materials, experimental systems and methods used in many studies. Here, indicate whether each material, system or method listed is relevant to your study. If you are not sure if a list item applies to your research, read the appropriate section before selecting a response.

Materials & experimental systems

n/a	Involved in the study	
☒	☐	Antibodies
☒	☐	Eukaryotic cell lines
☒	☐	Palaeontology
☒	☐	Animals and other organisms
☐	☒	Human research participants
☒	☐	Clinical data

Methods

n/a	Involved in the study	
☒	☐	ChIP-seq
☒	☐	Flow cytometry
☒	☐	MRI-based neuroimaging

Human research participants

Policy information about studies involving human research participants

Population characteristics	For human monocyte data generated by us, monocyte preparation uses a modification of published protocols. Briefly, blood (~20 ml for both 10x Genomics and SMART-Seq2 analysis) drawn in sodium heparin is processed immediately (lab in the Clinical Research Center). PBMCs are isolated by gradient Ficoll paque centrifugation, which maintains cell viability and prevents ex vivo activation during cell recovery. Cells are stained with antibody against human HLADR, CD14 and CD16103 and monocyte subsets defined as HLADR+CD14++CD16-(classical), HLADR+CD14++CD16+ (intermediate), HLADR+CD14dim/CD16++ (nonclassical, patrolling monocyte). DAPI staining is used to exclude dead cells. Monocytes are sorted by a BD Influx Sorter into tubes for real-time 10x Genomics analysis. And samples were sequenced at three different time points, so there are three batches (T1 for 01/08/2017, T2 for 17/10/2017 and T3 for 20/11/2017).
Recruitment	The corresponding clinical information was collected by a clinical coordinator at Columbia University.
Ethics oversight	IRB approval from Columbia University.

Note that full information on the approval of the study protocol must also be provided in the manuscript.
